# Supplementary material for: The influence of decision-making in tree ring-based climate reconstructions
Source: Nat Commun. 2021 Jun 7;12:3411. doi: 10.1038/s41467-021-23627-6 (PMC8184857; doi:10.1038/s41467-021-23627-6)
Supplement: Supplementary file 1 — Supplementary Information [file 41467_2021_23627_MOESM1_ESM.pdf]

Supplementary Information for "The influence of decision-making in tree ring-based climate reconstructions" by Büntgen et al. (2021) *Nature Communications*

Ensemble reconstructions R1–R15

Group 1 reconstruction (R1). R1 did not apply any selection criteria to the nine raw TRW datasets, because it assumes that freely available TRW data are correctly cross-dated by their initial providers, and that cross-dating without metadata is generally much more challenging. For each of the nine individual TRW datasets, R1 developed 16 different TRW chronologies based on slightly varying Regional Curve Standardisation treatments (RCS)[1-3]. The different approaches include varying degrees of filtering of the regional curve (i.e., secondary smoothing of the RC), in combination with variance stabilization[4], temporal data splitting and index calculation (i.e., ratios or residuals after power transformation)[5]. Moreover, R1 used both the Standard (STD) and Arstan (ARS) chronology versions from the latest generation of the ARSTAN software[6], with the latter normally containing lower first-order autocorrelation structures[7]. Each of these chronology development techniques was applied on all TRW series per site (all), and separately on the living and relict series per site (liv/rel). The resulting RCS chronologies of the split datasets were merged at equal weight when sample size exceeded 20 series[8]. Hence, R1 developed 16 different TRW chronologies (1–16): 1) all_ptRCS-10_STD (using RCS on all data, calculating residuals after power transformation, smoothing the RC with a length-adaptive -10y cubic spline, and taking the STD chronology values), 2) all_ptRCS-10_ARS (using RCS on all data, calculating residuals after power transformation, smoothing the RC with a length-adaptive -10y cubic spline, and taking the ARS chronology values), 3) all_RCS_STD (using RCS on all data, calculating ratios, and taking the STD chronology values), 4) all_RCS_ARS (using RCS on all data, calculating ratios, and taking the ARS chronology values), 5) all_RCS-10_STD (using RCS on all data, calculating ratios, smoothing the RC with a length-adaptive -10y cubic spline, and taking the STD chronology values), 6) all_RCS-10_ARS (using RCS on all data, calculating ratios, smoothing

the RC with a length-adaptive -10y cubic spline, and taking the RST chronology values), 7) all_sfptRCS10 (using signal-free RCS on all data, calculating residuals after power transformation, and smoothing the RC with a length-adaptive -10y cubic spline), 8) all_sfRCS10 (using signal-free RCS on all data, calculating ratios, and smoothing the RC with a length-adaptive -10y cubic spline), 9) liv/rel_ptRCS-10_STD (using RCS separately on the living and relict data, calculating residuals after power transformation, smoothing the RC with a length-adaptive -10y cubic spline, and taking the STD chronology values), 10) liv/rel_ptRCS-10_ARS (using RCS separately on the living and relict data, calculating residuals after power transformation, smoothing the RC with a length-adaptive -10y cubic spline, and taking the ARS chronology values), 11) liv/rel_RCS_STD (using RCS separately on the living and relict data, calculating ratios, and taking the STD chronology values), 12) liv/rel_RCS_ARS (using RCS separately on the living and relict data, calculating ratios, and taking the RCS chronology values), 13) liv/rel_RCS-10_STD (using RCS separately on the living and relict data, calculating ratios, smoothing the RC with a length-adaptive -10y cubic spline, and taking the STD chronology values), 14) liv/rel_RCS-10_ARS (using RCS separately on the living and relict data, calculating ratios, smoothing the RC with a length-adaptive -10y cubic spline, and taking the ARS chronology values), 15) liv/rel_sfptRCS10 (using signal-free RCS separately on the living and relict data, calculating residuals after power transformation, and smoothing the RC with a length-adaptive -10y cubic spline), and 16) liv/rel_sfRCS10 (using signal-free RCS separately on the living and relict data, calculating ratios, and smoothing the RC with a length-adaptive -10y cubic spline). Since R1 could not agree on a universally accepted, objective criterion for selecting a single best chronology version, it calculated the median of the 16 chronologies, which further contributed to variance stabilization in the resulting regional time-series over the past two millennia. The minimum and maximum chronology values of each year were considered as methodological chronology error limits[9]. Since R1 could not decide on a single best climatological product and season, it correlated the nine regional chronology medians against current-year, average June, July or August instrumental temperatures averaged from the nearest 0.5° × 0.5° CRU TS4.03 and 1.0° × 1.0° Berkeley grid

cells[10,11]. R1 decided to restrict all growth-climate response analyses, and thus proxy-target correlations to the post-World War II period for which the gridded climate indices in all regions are based on a markedly expanded and improved network of climatological station readings[12], and for which each of the individual TRW chronologies is composed of hundreds of TRW samples. R1 then averaged data from the latest CRU and Berkeley versions because they are nearly identical ($r > 0.98$), and there is neither a statistical nor methodological and conceptual justification to prioritize one over the other. R1 considers proxy-target correlation coefficients > 0.4 as highly significant after correction for first-order autocorrelation ($p < 0.01$). Since the two mid-latitude TRW chronology medians from the Great Basin (GTB)[13] and the Southern Colorado Plateau (SCO)[14] in the western United States do not contain a sufficiently strong temperature signal between 1950 and present, likely affected by unprecedented anthropogenic drought stress during the past decades[15], these two datasets were excluded in any further steps. All of the remaining seven ensemble medians (QUL, NSC, ALP, YAM, TAI, ALT, NYA), however, reveal significant positive correlation coefficients ($r > 0.4$; $p < 0.01$) with current-year, average June, July or August temperatures over the 1950–2002 CE period of proxy-target overlap. R1 then scaled the seven TRW chronology medians that exhibit highly significant correlation coefficients with regional June, July or August temperatures against their best summer season temperature targets using the mean of the nearest CRU and Berkeley grid points[10,11]. R1 used scaling instead of regression to avoid artificial variance reduction[16]. After scaling at the regional-scale, the median of the seven regional medians was used to reconstruct large-scale summer (JJA) temperatures from 1–2010 CE, which is the common period of all remaining regional TRW medians. R1 restricted the NH reconstruction to the extra-tropics > 30°N between 180°W and 180°E, and used the accumulated error bars from the regional TRW chronologies (using the minimum and maximum values per year from each of the 16 chronology versions), as well as the Root Mean Squared Error (RMSE) from scaling against the gridded regional summer temperatures[9].

Group 2 reconstruction (R2). R2 did not apply any selection criteria to the nine raw TRW datasets. With a primary aim of capturing as much secular scale variability from the data as possible, RCS was employed. All TRW data were power transformed prior to detrending (via subtraction), while chronology variance was stabilised as a function of changing sample size through time[4]. The TRW data for each site were combined and sub-sampled for RCS detrending using two different approaches: i) RCS3grp – the data were split into three groups of different growth rates – low, medium and high. RCS was performed (using signal free) separately on each group and the final detrended group data averaged to create a composite chronology. Detrending was performed using the CRUST software[3]; ii) in mRCS multiple RCS groups were created using 'Growthv36beta' by splitting the data into 12 sub-groups with respect to different classes of growth rate and age. RCS was performed on each sub-group (no signal free) and the resulting indices averaged to create the final composite chronology. Each of the nine input TRW chronologies were truncated to exclude periods represented by less than ten series. To create the final NH composite reconstruction, each site chronology was standardised to z-scores with respect to the 1000–1850 period, and four different compositing approaches were used: i) A simple average (no spatial weighting) of the nine regional chronologies, adjusting variance in the final mean as a function of changing number of series through time. ii) Equal weighting of North America and Eurasian data, again adjusting variance in the continental and final NH mean series as a function of changing sample size. iii) A weighted average of the nine regional TRW chronologies using the data-specific running 30-year Expressed Population Signal (EPS)[17] values as the weighting term. iv) Equal weighting of low and high latitude data; adjusting variance in the final mean as a function of changing sample size. Sites defined as lower latitude were: GTB, SCO, ALP and ALT. With the two RCS approaches and the four spatial averaging methods, R2 calculated an ensemble of eight slightly different NH composites were derived. Each NH composite series was scaled (same mean and variance) to CRUTEM JJA mean temperatures over the period 1880–1980 CE, and averaged together using the r-square (R^2) relationship with CRUTEM as a weighting function. Two error terms were calculated and combined. Firstly, the RMSE was calculated over 1880–2009 (purposely

including the post-1980 period) for each NH variant. This portrays the calibration (plus validation for the post-1980 period) error. There is substantial divergence in the recent period so including the post-1980 period increases the uncertainty range. R2 used the maximum RMSE values for each year to derive a calibration-based error for the final weighted NH composite. A further error term was generated by using the standard deviation between the eight scaled composite versions for each year to capture the detrending error related to the different variants. The calibration RMSE error and detrending error were then combined and doubled to give the final 2-sigma error for the weighted NH reconstruction.

Group 3 reconstruction (R3). R3 did not apply any selection criteria to the nine raw TRW datasets. R3 used the RCS approach to develop TRW chronologies that have the potential to preserve low-frequency information[1,18-19]. This method was supplemented by the signal-free approach and the calculation of multiple RC curves. The former was designed to mitigate possible limitations related to the trend-in-signal bias usually seen at the start and end of chronologies, whereas the latter approach was designed to mitigate limitations related to differing contemporaneous growth rates[19,20]. R3 calculated all nine TRW chronologies based on all TRW series using the CRUST software[3]. For each chronology, R3 used four sub-RCS curves (divided as four different tree growth-rate classes) and the signal-free approach with a maximum number of iterations of ten. Furthermore, R3 used the age-dependent smoothing approach to filter the RC curves, and stabilized the variance in the final chronologies[4]. The EPS and average correlation between series (Rbar) were also used to measure the strength of the 'expressed' chronology signal[17]. R3 only used the chronology interval with an EPS value larger than 0.85 to produce the final composite reconstruction. R3 then applied a nested principal component regression (PCR)[21] approach to the nine TRW chronologies to conduct the warm-season (May–September, MJJAS) mean temperature reconstruction for the NH. R3 used the mean of the MJJAS 30–75°N temperature anomalies (with respect to 1961–90 CE) over land from the CRUTEM4 dataset[10] as reconstruction target. This approach created a suite of nests, considering that the number

of available TRW chronologies decreased before the earliest common year 208 CE and after the latest common year 2002 CE of the nine regional TRW chronologies. R3 used a sliding window approach for calibration (using 2/3 length of instrumental data over the 1880–2002 CE) and verification (using 1/3 length of instrumental data over the 1880–2002 CE) to produce the final reconstruction[22,23]. In each nest, the initial calibration interval extends from 1880–1961 CE, and was incremented by one year until reaching the final interval 1921–2002 CE, creating an ensemble of 41 reconstruction members. The reduction of error (RE), coefficient of efficiency (CE), root mean square error (RMSE), and r-square (R^2) statistics were used to assess the skill of each nested model[24]. The temperature reconstruction, RE, CE, r-square (R^2) and RMSE values were expressed as the ensemble median of the 41 members. Finally, the most replicated nest, i.e., the one from 208–2002 CE, was scaled to have the same mean and variance as the instrumental target over the period 1880–2002, and other nests were scaled to the most replicated one. The relevant time-series sections of each nest with no less than five chronologies were spliced together to derive the full-length reconstruction covering the period 1–2015 CE.

Group 4 reconstruction (R4). R4 applied a selection criterion based on the correlation between series, and removed those series that presented either negative correlations (including at least 30 years of data), or very low correlations ($r < 0.2$). R4 developed four different TRW chronologies based on slightly varying RCS treatments. The different approaches include varying degrees of filtering of the regional curve (i.e., secondary smoothing of the RC), in combination with variance stabilization[4], temporal data splitting and index calculation, such as ratios or residuals after power transformation[5]. Moreover, R4 used both the Standard (STD) and Arstan (ARS) chronology outputs from the latest version of the ARSTAN software[6], with the later generally containing less first-order autocorrelation[7]. Each of these chronology development techniques was applied on all TRW series per site (all). The resulting RCS chronology versions of the split datasets were merged at equal weight when sample size exceeded 20 series. Hence, R4 developed four different TRW chronologies (1–4): 1) all_ptRCS_STD

(using RCS on all data, calculating ratios after power transformation, and taking the STD chronology values), 2) all_ptRCS_ARS (using RCS on all data, calculating residuals after power transformation, and taking the ARS chronology values), 3) all_RCS_STD (using RCS on all data, calculating ratios, and taking the STD chronology values), 4) all_sfptRCS (using signal-free RCS on all data, calculating ratios after power transformation, and taking the STD chronology values. R4 correlated the regional chronologies against current-year, average June, July or August instrumental temperatures averaged from the nearest 0.5° × 0.5° CRU TS4.03 grid cells[10]. R4 decided to restrict the growth-climate response analysis, and thus proxy-target correlations to 1902-2014 period for which each of the individual TRW chronologies is composed of hundreds of TRW samples. R4 considered proxy-target correlation coefficients > 0.4 as highly significant after correction for first-order autocorrelation ($p < 0.01$). Since the two TRW chronologies from northern Yakutia (NYA)[25] and Taimyr (TAI)[26], did not contain a sufficiently strong temperature signal between 1902 and present, or portray a stronger signal with other climatic variables (i.e., precipitation or drought indices), these two datasets were excluded in any further steps. All of the remaining seven chronologies (GTB, SCO, QUL, NSC, ALP, YAM, ALT), however, reveal significant positive correlation coefficients ($r > 0.4$; $p < 0.01$) with current-year, average June, July or August temperatures over the 1904–2014 CE period of proxy-target overlap. R4 selected the chronology from each site with the best growth-climate correlation (in all cases the best performance was given after either 'all_ptRCS_STD' or 'all_sfptRCS'), and then regressed the mean of the chronologies. R4 calculated the robust average that is less affected by outliers, between the individual detrended chronologies to compute one final chronology. R4 reconstructed large-scale summer (JJA) temperatures from 1–2010 CE, which corresponds to the common period of all seven regional TRW chronologies finally used. R4 restricted the NH reconstruction to the extra-tropics > 30°N and between 180°W and 180°E, and used the Root Mean Squared Error (RMSE) from regressing against the gridded regional summer temperatures.

Group 5 reconstruction (R5). R5 noted a large number of very short TRW series for several sites, and dealt with those in two passes. First, very short series (< 40 years) were removed from all regional data files due to concerns about how very rapid growth might distort the final TRW chronology (despite standardization procedures). This was considered particularly relevant when, for some sites (most notably ALP), many of the very short series were from living trees that would form the modern end of the resultant chronologies. As R5 has previously found that available TRW data may still contain dating issues, cross-dating within each site file was (re)checked using the COFECHA software. Any TRW series with a correlation of < 0.3 with the site master chronology was examined in more detail. A number of TRW series were discarded due to very poor correlations with the master chronology for periods with reasonable sample depth (≥ 25). However, if individual TRW series had low correlations due to only one very poorly correlated segment, they were not removed from the regional files. Although the sheer numbers of samples for each site meant that these were unlikely to have impacts on the final series, R5 preferred to use 'clean' files in any further step. A total of three series across all sites displayed very obvious dating errors across the whole sample and these were corrected by shifting the whole series. A typographical error in the GTB series was also corrected. At this point, R5 conducted a second pass for short series. The definition of 'short' was now related to the typical lengths of series at each site. SCO and GTB typically consisted of very long TRW series, so only series of < 100 years were removed; for ALT, series of < 70 years were removed; for QUL and YAM, series of < 40 years were removed. For the remaining sites, series of < 50 years were removed. However, short individual TRW series covering periods of very low sample depth (< 25) were retained in each dataset. The final chronologies therefore included the following number of series: ALP = 1788, ALT = 736, GTB = 721, NYA = 1799, QUL = 2594, SCA = 1126, SCO = 224, TAI = 349, and YAM = 655. In deciding how to standardize series, R5 first visually examined the TRW series of each site, finding that many, across all sites, exhibited declining growth with age. Some studies have identified RCS as being unsuitable for Bristlecone pine (GTB and SCO) due to distorted growth patterns as well as issues around estimating pith offset[14]. Therefore, negative exponential curves were used to

standardize series from these two sites. Indices were computed using power transformed residuals[5]. For other sites, R5 used multiple RCS curves. Several studies for several sites commented on, or alluded to, the ability to estimate pith offset or careful sampling to capture pith[27-29]. Data were split into groups based on the age spread at a site, and growth rate. The number of series per class was also a consideration. All indices produced were based on power transformed residuals rather than ratios[5]. Signal-free detrending aims to maintain middle frequency variance related to climate variability and has been shown to reduce end-series distortions in many cases[4]. All TRW series were processed in the signal-free framework. However, distortion of the ends of series has been reported in some cases, so the standardized version of series was compared with those subjected to signal-free processing. The ALP data had obvious end distortion effects the standard chronology was utilized for this site. Signal-free chronologies for all other sites were produced. In order to select a target season for reconstruction, R5 examined the local seasonal signal of the different chronologies across small regions around the sites. For this purpose, R5 used the 1° × 1° gridded Berkeley Earth anomaly data[11]. Average monthly correlations across surrounding grid squares (number varying depending on site extent) were calculated, and from these, several potential reconstruction seasons for the warmer months were identified. These seasons included June–August, July–August, June–July and July (JJA, JA, JJ and July). The relationship across each grid square in a slightly larger area surrounding the site was then examined to obtain an idea of signal fidelity of each potential season on this regional scale. Based on results for both an 'early' (1900–1950 or 1900–1940 (YAM) and a 'late' (1951–2000 CE, varying slightly due to divergence issues – see below), the JJ season was considered optimal across sites. R5 then plotted the averaged JJ temperature series across the relevant region against the chronology (from 1900 to end of the chronology) to visually check for any obvious signs of divergence. Several sites showed a clear divergence at the modern end of series (most notably in TAI and ALT). Data after 2000 were therefore not included in the calibration or verification periods in the subsequent reconstruction in an effort to avoid any distortions this might introduce. To test the ability of the 9 chronologies to capture variability in NH temperature, R5 opted to use the CRUTEM4.0 NH data[10] averaged anomaly

series for JJ as the target (0–90°N and 180° W to 180°E), noting that the chronologies are all located in the mid- and high-northern latitudes, and hence that signal strength is likely compromised. Further, R5 used a simple principal component regression approach for reconstruction in which all chronologies, and lag-1 chronologies were forced into the analysis. Lag-1 chronologies were used because preliminary analyses indicated statistically significant associations with summer temperature in the prior season (ALP, NYA, QUL, TAI, SCO, and YAM) Autoregression was modelled for both the climate and TRW series. No weighting applied to predictors, and this, along with their all being forced into the analysis, may present a further challenge to signal strength. Variance in the reconstruction was stabilized through the use of a 400-year spline. A split calibration-verification period scheme was used in which the 1901–2000 CE period was used for calibration to try to capture as much variability in the model calibration period as possible. The 1850–1900 CE period was set aside for verification, even though there is less data available for this period; yet another potential challenge for this reconstruction. The resulting reconstruction covered the 1–2000 CE period. Error estimates were calculated in two ways; first using 300 bootstrapped reconstructions, and second, through making use of the bootstrapped RMSE values. R5 considered this reconstruction to be minimalist in its sophistication.

Group 6 reconstruction (R6). R6 excluded all data from the two sites GTB and SCO, since those did not reveal a sufficiently strong temperature signal. R6 therefore included only seven regional TRW chronologies in the final large-scale NH reconstruction (QUL, NSC, ALP, YAM, TAI, ALT and NYA). For standardisation, R6 used the RCS SSF approach[2,30] via the open access software (RCSigFree_2018_Win) with the following settings: indices by ratios, power transformation, age-dependent spline smoothing, robust Tukey bi-weight mean, and no pith offset estimates. R6 then normalized the individual site chronologies over their common period (1–2010 CE), and combined them to provide an overview of variations in tree growth. For further reconstruction purposes, R6 simply averaged the seven normalized TRW chronologies, because all of them expressed positive

correlations between each other. On the basis of site and mean growth-climate response analyses, R6 used JJA mean temperatures as the target season for climate reconstruction. Since it was difficult to test if the climate signal in the seven TRW chronologies was constant over time, R6 decided to not use multiple regression or weighted averaging, but conclude that the simple average of the seven normalised TRW chronologies invokes the fewest assumptions though still provides a reasonable correlation with the target season. R6 averaged gridded CRU TS4.04 data over the sevven regions, where spatial correlation coefficients of the site TRW chronologies against gridded JJA temperatures were significant at p = 0.1. The final target grid boxes varied between 0.5–10° latitude and 2.5–10° longitude, and the arithmetic mean of all grid boxes (from the seven regions) was calculated as anomalies with respect to the 1961–90 reference period. R6 used the +/- 2 standard error as reconstruction uncertainty, which is probably an underestimation of the 'true' error range.

Group 7 reconstruction (R7). R7 detrended all individual series of all nine TRW datasets using the RCS method at a chronology-specific scale. R7 applied a spline with a 50% cut-off to the RCS curve in the R dplR package[31]. Due to a lack of pith offset data, R7 arbitrarily set the pith offset data for each series as (cambial) year 1. R7 selected a subsample sample strength (SSS) threshold of ≥ 0.85 to truncate each chronology[32]. No individual TRW series was removed from any of the originally published regional chronologies. To select a temperature target for reconstruction, R7 used monthly temperature anomaly (relative to 1961–90 CE) data of the gridded (5° × 5°) CRUTEM.4.6.0.0 (1900–2017 CE) data set. Based on the field correlation and climate response in the original publications, as well as the geographical distribution of the chronologies, R7 used temperature data for the extratropical NH (NH; 30–90°N) as potential reconstruction target. R7 averaged monthly temperature anomalies over the NH domain to obtain potential monthly temperature targets. R7 then calibrated each TRW chronology against the monthly and seasonal NH temperature anomaly time-series (1900–2017 CE). Based on the most common of these climate responses, R7 selected May through September as the seasonal target for reconstruction. R7 also examined the coherent variability among the nine

chronologies using Pearson correlation analysis over three periods (1–2016; 1000–2016; and 1800–2016 CE). Of the nine regional chronologies, R7 selected five chronologies to include in our NH temperature reconstruction based on three criteria: 1) statistically significantly positively correlated with summer (May–September) NH temperature anomalies (excluding TAI); 2) showed significant correlation with the other chronologies (eliminating NYA and QUL), and 3) equal weight in geographical distribution, meaning only one chronology, with the stronger temperature signal and the longest reliable period, was included in the final reconstruction per region, even if two or more chronologies were available (eliminating SCO). Five temperature-sensitive regional TRW chronologies (ALP, ALT, GTB, SCA and YAM) were selected in our final reconstruction. R7 used a nested principal component analysis (PCA) approach[33] to reconstruct NH summer temperature. The PCA method can reduce the input data matrix to a few component scores and can address the common coherent variability in the proxies[33] without a large loss of signal and has been used in large-scale reconstructions[33,34]. R7 applied the PCA analysis based on variance maximize rotation using the R package 'psych' for three nesting periods (at least four chronologies in each nest)[35], which were determined by the length of the common period among chronologies with SSS ≥ 0.85. To produce each PC nest, R7 selected the first principal component (PC1), which accounted for more than 40% of the total variance for each of the three nests. R7 examined the strength of the temperature signal in each PC nest using Pearson's correlation coefficients between PC nest and potential monthly and seasonal temperature targets and selected the May–September temperature anomaly as our reconstruction target because it was the most strongly correlated with each of the PC nests. Finally, R7 applied backward nest and forward nest PCA reconstruction procedures to merge the three nests. R7 used split-period calibration and verification tests to determine the reconstruction skill of each PC nest against two sub-period of the CRU NH May-September temperature target (1900–1955 and 1956–2010 CE). R7 assessed reconstruction skill using the statistical parameters of the reduction of error (RE) and coefficient of efficiency (CE)[36]. R7 estimated the uncertainty of the reconstruction based on the calibration uncertainty[37], which R7 expressed as root mean square error (1 RMSE) derived from

the linear regression of each PC nest against the temperature target. The range of 1 RMSE was added to the reconstruction as uncertainty intervals for each PC nest.

Group 8 reconstruction (R8). R8 applied no selection on the individual TRW chronologies, except one based on the length. The TRW series of length less than 100 years are removed. For each of the nine sites, the ARGC method[38] is applied to standardize the individual TRW series and to calculate a master TRW series. The standardisation is based on the biological growth trend, which tries to remove the trend linked to the age of the tree. The process is adaptive in that way that for each tree, the trend is based on the regional curve approach but modified according to the initial and the maximum growth rates (measured by the TRW increments) of each tree. R8 used an artificial neural network to estimate a common relationship between each TRW and three predictors: the age of the ring, the initial (mean of the first 10 rings) and the maximum growth calculated on the juvenile period set here on 50 years. When the squared-R of the so calculated trend explains a variance inferior to the mean growth (on the whole series), the standardization is done by smooth curve (loess function from R-package MASS). The indices are defined as the ratio between the TRW and its corresponding trend. For the temperature data, R8 used the land temperature dataset CRUTEM.4.6.0.0.anomalies.nc provided by the Climate Research Unit[10]. It contains time-series from 1850–2019 by grid points spaced by 5° of longitude and latitude. The NH data from 30–90°N are selected. Only the time-series larger than 50 years are kept, such as 452 series from a total of 864. The missing data are imputed using the fill.SVDimpute function of the filling R-package. The JJA mean temperature is chosen as this variable is the most commonly used in the nine initial papers describing the TRW data. A principal component analysis of the 452 retained gridded JJA series show that the first principal component explains 6% of the total variance and is closely correlated with the NH mean. R8 used the mean NH (> 30°N) JJA temperature as variable to be reconstructed, denoted T_JJA_NH. It covers the 1850-2019 period. As a first check, a regression between T_JJA_NH and the nine TRW chronologies is calculated. The squared-R is 0.23. R8 used the analogue method based on a decomposition of the series in low and high frequencies[39] to

perform the final climate reconstruction. The proxies and climate series are decomposed into two frequency bands by using two complementary filters: high frequency (HF, with frequencies f > 0.2), and low frequency (LF, f < 0.2). The reconstruction of T_JJA_NH in each frequency band is based on the analogues defined respectively on the HF and LF proxy series, respectively. The AM is applied to extrapolate the temperature series on the common era in both frequency band. R8 used a block Jackknife method to verify the reconstruction. It simulates 50 subsets of data by randomly taking observations in the common time-period (1850–2002 CE). For each of the 50 simulations and for the common period: 1) An observation is randomly drawn for independent verification and the three (one) neighbour(s) for LF (HF) respectively, on each side, are excluded as potential analogues, to avoid overestimation due to autocorrelation. 2) Also, to avoid autocorrelation problems in the LF band, one observation is randomly taken by blocks of three for the possible analogues, for the HF band, all the not drawn observation may be used for analogues. 3) the climate of all the observations is estimated using the closest one from the possible analogues (Euclidian distance). Points 1 to 3 are repeated 50 times. At the end, R8 obtained 50 observations for independent verification (prediction statistics) and statistics calculated on the other observations are calibration statistics. The two frequency bands are recombined by summation and provide estimates of full spectrum of T_JJA_NH. Temperature estimates from the randomly taken years are considered for an independent validation. Comparisons of these 50 estimates with the observed temperature from the included years provide calibration statistics. The reconstructions are then summarized into median and 95th percentile confidence intervals. On the LF band, the squared-R (calibration) and the RE (verification) are respectively 0.76 and 0.71 and on the HF band, they are respectively 0.55 and 0.75. The final squared-R (HF and LF recombined) is 0.72 and the RMSE is 0.17°C

Group 9 reconstruction (R9). R9 applied no selection criteria on the nine raw TRW datasets. It assumes that freely available TRW data are correctly cross-dated by their providers, and that cross-dating without metadata is not possible. For each of the nine individual TRW datasets, R9 developed

nine detrended TRW chronologies by fitting a modified negative exponential curve (hereafter referred to as NegExp) representing a classic nonlinear model of biological growth to individual series of tree measurements. If that nonlinear model cannot be fitted, then a standard linear model was fitted. Dimensionless indices were obtained by dividing the observed TRW data by the value predicted by the NegExp or the linear model. Growth indices were averaged for each TRW dataset by year using a Tukey's biweight robust mean which minimizes the influence of outliers using the DPLR package in R 4.0.2. R9 used a principal component regression (PCR) approach to transfer TRW data into NH summer (JJA) temperature units, expressed as anomalies with respect to the 1961–90 CE reference period. A reduced space signal of the proxy records was then extracted using principal component analysis (PCA), resulting in a set of principal components (PC) and PC loading patterns. The first n PCs with eigenvalues > 1.0 were retained as predictors to develop a multiple linear regression model. A multiple cross validation using random calibration sets (bootstrapping) was applied to the PCR to estimate the skill of the reconstruction and confidence intervals around the reconstructed anomalies. Because each chronology length differs, an iterative nesting method was used to develop the temperature reconstruction. This procedure entails the sub-setting of the original dataset into complete data matrices without missing values, so-called nests. In total, six nests have been adjusted to the common period of all series. The nested PCR was computed schematically following a three-step procedure. In each nest, firstly, the number of predictor variables was reduced using a principal component analysis; secondly, the PCs with eigenvalues >1 were retained as independent variables within Ordinary Least Square (OLS) multiple regression models while a mean NH JJA temperature series (40–90°N), obtained from the Berkeley Earth Surface Temperature (BEST) gridded (1° × 1° latitude/longitude) dataset[11] over the period 1801–2002 CE, was used as a target. The robustness of each model was tested based on a traditional split calibration/verification procedure bootstrapped 1000 times and the final reconstruction of each nest was computed as the median of the 1000 realizations, given with their 2.5–97.5 percentiles. The skill of each reconstruction has been evaluated based on (i) the coefficient of determination (r^2 for the calibration and R^2 for the verification periods), (ii) reduction

of error (RE) and (iii) coefficient of efficiency (CE) statistics. The final reconstruction (0–2016 CE) was achieved by splicing all the nested time-series after the mean and variance of each nested reconstruction segment had been adjusted to the best replicated nest (0–2002 CE).

Group 10 reconstruction (R10). R10 was motived by a concern for how uncertainty in the mean value of individual series accumulates backwards in time, as necessitated by the fact that individual series span a small fraction of the total interval of the reconstruction. In the most extreme case (YAM), the total record period is more than twenty times as long as the average core length and more than eight times as long as the longest individual core record. Thus, no common calibration period exists. Simulations indicate that the uncertainty in aligning overlapping sections leads to accumulated uncertainty in the mean value of subsequent sections that can come to dominate the low-frequency variability of the inferred climate history. To better control uncertainty in overlapping segments, R10 developed an iterative method to minimize the least-squared difference between pairs of estimates for each year across all overlaps between a series of cores by adjusting the unknown mean value of each series. The computational cost of our implementation grows rapidly with number of records. In practice, R10 used 200 cores per site, which requires approximately an hour of computation per site. The 200 cores are selected both according to number of data points and evenness of coverage. For each site, R10 first selected the longest single record and next that record with the most years not sampled by the first core, continuing on in this manner until all years are sampled at least once. R10 then picked cores that had the most record years not yet sampled twice and so on until a total of 200 cores are selected. All further analysis involves only this subset of 200 cores per site. A more efficient procedure for determining optimal mean values is likely possible and would permit for selecting more cores. Our approach to pre-processing each of the 200 cores is simple and standard. Each core is detrended by first power transforming and then fitting and subtracting a spline curve with a rigidity such that 50% of the signal passes at a length equal to 2/3 of the core record length. R10 recognized that methods have been designed to preserve more low-frequency signal than spline detrending, but R10 was

concerned that such methods may be prone to larger error variance in the very early portion of the record, and that such error may then lead to inflated low-frequency variability when many records are daisy-chained across an interval much longer than the length of individual cores. After detrending, R10 scaled each series to unit variance and calculate the mean value for each series that minimizes the global-squared error between each pair of common estimates, as described in the foregoing paragraph. A single chronology is obtained at each site by averaging across all cores for each year between 1 and 1970 CE. Each chronology is variance-scaled to match JJA average temperatures from the closest time-series in the Gridded Berkeley Earth Surface Temperature Anomaly Field (version 16-Jan-2020), with the mean of the TRW chronology set to zero over the common calibration period. A statistically significant correspondence ($p < 0.01$) between the chronology and nearest instrumental temperature record is found at all nine sites. Therefore, all sites are retained in computing a large-scale average. A large-scale reconstruction was obtained simply by averaging the individual reconstructions from each of the nine sites. For purposes of comparing against the trees-only reconstruction, a thermometers-only reconstruction was also obtained by setting the mean of nine thermometer time-series (taken from closest grid boxes to sites) to zero over a common calibration period and averaging. The combined-reconstruction represents the average of the trees-only reconstruction and the thermometers-only reconstruction over their period of mutual overlap.

Group 11 reconstruction (R11). R11 applied no selection criteria to the raw TRW series, as it was assumed that the datasets were properly cross-dated and quality checked by the data providers. However, a handful of duplicate measurements (< 10 series) were removed prior to analyses. The standardization was performed site-by-site in MATLAB Version: 9.5.0.1033004 (R2018b), adopting a multiple (2-curves) RCS method[19]. The TRW data were first aligned by ring age. The alignment was based on the earliest date of each tree core, as no pith-offset estimates were available for the material. A simple average was calculated on the aligned series. Note however that where the age-aligned replication dropped below 20 series, the data average was replaced with a mean of the previous 50

years to the drop below 20 samples. A 100-year cubic smoothing spline was then fit to the resulting mean curve. Subsequently, data were separated into two cohorts based on the RCS curve: a fast-growing cohort, a mean of the TRW measurement > mean of the RCS curve (for the period of overlap between the RC and each measurement) and a slow-growing cohort (mean of the TRW measurement < mean of the RCS curve (for the period of overlap between the RC and each measurement). The procedure was then repeated for the two cohorts separately, where separate RC's were created for the faster- and slower-growing samples. Individual TRW measurements were then divided by the corresponding RC to produce TRW indices. The final chronology was produced as an arithmetic mean of all indices from both cohorts. The variance adjusted CRUTEM4v data[40] surface temperature was utilized for growth-climate response analysis. To assess the characteristics and strength of the site temperature response of the individual predictor series, R11 first performed calibration experiments between individual TRW records and local/regional temperatures (mean of all 5° × 5° longitude-latitude grid points within each sampling region). The calibration was conducted over a number of periods (e.g., post-1950, post-1900, post-1850), using both the original as well as first-differenced data. After exploring the local temperature response, R11 also evaluated the sensitivity of the TRW records to large-scale temperature variability. All nine chronologies were determined to possess useful information, and were therefore normalized (z-scored) over their common period and then averaged, given equal weight to each individual series, into a composite chronology spanning the 1–2010 CE period. The composite chronology was then calibrated against area-weighted, extratropical NH (bounded by coordinates 30–70°N and 180°W to 180°E) average JJA temperature. Collectively, these assessments identified mean JJA average temperature as the most optimal instrumental target for reconstruction. This selection was based on a trade-off between the length of the season and the median correlation coefficient between the temperature and all the predictor time-series. The composite chronology revealed a significant positive correlation with JJA NH temperatures over the 1850–2010 period, both based on original ($r = \sim 0.6$; $p < 0.05$) and first-differenced ($r = \sim 0.3$; $p < 0.05$) TRW and temperature series. For the final reconstruction, R11 therefore utilized the composite chronology

derived from all nine sites as the predictor series, and the area-weighted extratropical NH JJA temperature averages as the reconstruction target (predictand). A split period (1850–1930, 1931–2010) calibration/verification procedure was used to assess the skill of the reconstruction, whereas the final model was built over the entire 1850–2010 period. Statistics that were used to evaluate the quality of the estimates included R^2, R^2 adjusted, RE and CE statistics. The final reconstruction, covering the period 1–2010 CE, was scaled to the instrumental data over the 1850–2010 period. The reconstruction uncertainty was estimated by RMSE, based on the residuals between actual temperature and scaled estimates.

Group 12 reconstruction (R12). R12 started from the standardization of the individual TRW series. Age band decomposition (ABD)[41] was explored in removing the age-dependent growth variability from the TRW data representing the nine preselected chronologies/sub-regions. This method (ABD) was used since it removes the effect of tree age from the original (raw) TRW series but preserves the other (presumably climatic) long-term, low-frequency variability in the resulting chronologies as originally demonstrated for a NH collection of TRW-chronologies[41]. The North American, European and Asian chronologies were considered representing three regions used for building the regional ABD series. A 10-year banding for tree rings up to 100 years old (ring count was used for estimating the biological tree age since no information of pith offset was available) was used to account for the faster rate of declining TRW in younger trees, and a wider banding of 50-years was used for tree rings representing older trees. The TRW series derived from rings within their bands were averaged within the sub-region they represent and the mean series were converted to z-scores. The z-score records were regionally averaged, the resulting mean series being converted to z-scores. The base period 1701–2000 CE was used for all the foregoing z-score calculations. Mean chronologies were calculated over the period when they contained at least eight series corresponding to intermittent minimum over the common period which accordingly represents the years 72–2002 CE. The minimum number of eight series was decided based on the fact that one of the chronologies contained less than ten series over

several pre-instrumental intervals with a transient eleventh century minimum of eight series. The target data for the reconstruction was the instrumental CRUTEM3v temperature dataset[42], expressed as temperature anomalies from the base period 1961–90 CE on a 5° × 5° grid-box basis[43]. A subset of data representing latitudinal band between 15–65°N and JJA season was used. This was the latitudinal band with strongest temperature correlations constantly over the sub-periods (1901–1951 and 1952–2002 CE). Calibration period was the 20th century (here, 1901–2002 CE). Linear regression ($R^2 = 0.43$) was used for substituting the proxy data with reconstructed temperature values. The reduction or error and the coefficient of efficiency were positive from the early (1901–1951) and late calibration (1952–2002) trials over the late (1952–2002) and early verification (1901–1951) data withheld from the respective calibrations, which is commonly taken to indicate that the reconstruction has some skill in reproducing the instrumental observations.

Group 13 reconstruction (R13). R13 applied no preselection to the raw TRW data. Whenever possible, R13 used existing detrended and standardized chronologies, following a philosophy that the collectors and developers of individual sites have the most direct knowledge of and experience with detrending and standardization of those species and locations. Publicly available and published chronologies were used for ALP, ALT, QUL, and SCO. This was particularly important for ALP and QUL, as both in the detrending procedure applied by the original authors was complex, and in the case of QUL more standard approaches can yield substantially different low frequency behaviour in the final chronology. The Tornetrask chronology[30] was used for SCA and the Yamal chronology[44]. R13 applied the same detrending to the GTB TRW data as used by[13]: Negative exponential, linear, or negative slope with power transform, residuals chronology calculation[5], and variance stabilization[4] and used the ARSTAN chronology. R13 used a single curve RCS with spline to develop the chronology for NYA and TAI. R13 tested the two-curve approach for TAI used by[26], but there was not sufficient metadata available and experiments yielded inconsistent and highly sensitive results. R13 evaluated the potential seasonal temperature signal at each individual site against the CRUTEM4.6

land-only dataset[45] and the kriged HadCRUT 4 temperature field[46] on monthly time steps applying both raw and first differenced data. Sites have varying seasonal responses. For instance, ALP appears to be unambiguously a JJA signal ($r > 0.40$), with the strongest signal in July and August, while the ALT is dominated by a June signal ($r = 0.57$). TAI and YAM are largely July ($r = 0.57$ and $r = 0.66$, respectively). Unsurprisingly, given considerable autocorrelation in the North American five needle pines[47,48], the interannual variability at GTB and SCO is not associated strongly with any individual month and the temperature signal appears to be stronger at lower frequencies[13]. R13 selected a JJA reconstruction target although also tested JJ, JA, and July alone as alternatives targets. All the European and Asian chronologies and QUL have local correlations with the JJA temperature field of $r > 0.40$, while GTB and SCO both have weak local interannual correlations which confounds high resolution reconstruction and skill over western North America. The final reconstruction target was the 40–75°N zonal mean between 180°W and 180°E from the CRUTEM4.6 land-only dataset[45,49]. R13 used a nested Composite-Plus-Scale (CPS) approach[49,50] to scale the average of the available TRW chronologies to the mean and variance of the zonal mean JJA temperature target and confined the period of the reconstruction to 1–2019 CE. R13 used a calibration period of 1930–1996 (the period of complete coverage of all chronologies) and validated against 1880–1929. R13 used an ensemble method where up to three of the nine available TRW chronologies were removed from the predictor pool to generate a 130-member ensemble reconstruction with varying predictor datasets and to observe the effect of including individual or groups of records, especially the low frequency dominated GTB and SCO. R13 identified reconstruction ensemble members that had $RE > 0.0$ for at least the last millennium and used these as a subset of the most skilful reconstructions (five of the 130). R13 also correlated the median of these reconstructions back against the HadCRUT4 field to evaluate the spatial patterns of reconstruction skill across the hemisphere. In addition to RE, uncertainties were quantified using the full spread of the 130-member ensemble and the Root Mean Square Error (RMSE) calculated for the calibration and validation periods.

Group 14 reconstruction (R14). R14 did not conduct any screening based on the length of individual TRW series, though excluded those series that had an inner date earlier than BCE 500. Site-level composite chronologies were generated via the signal-free implementation of Regional Curve Standardisation using the RCSigFree software (version 45v26)[3]. TRW measurements were converted to indices by computing residuals from the estimated growth curve and then power transformed[5]. The estimate of the RCS curves for each chronology was made using an age-dependent spline (with its initial stiffness set to ten years). The robust Tukey bi-weight robust mean was used to compute the mean chronologies, and its variance was stabilized by applying an age-dependent spline[4]. Chronologies were truncated to exclude the portion of the record when the Expressed Population Signal fell below the 0.85 threshold[17]. R14 identified the seasonal climate signal in each regional TRW chronology using the Seascorr program. For each site, the chronology was compared against local monthly temperature and precipitation values from Version 4.01 of the Climatic Research Unit's gridded climate dataset[51]. Most TRW chronologies (seven of nine) had highest correlations with mean temperatures from JJA or July–September. The two chronologies that did not exhibit a significant correlation with summer temperatures (SCO and NYA) were excluded from further analysis. The spatial correlation between each of the remaining seven TRW chronologies and mean JJA temperature was mapped across the NH, and based on the geographic patterns from those tests, R14 identified 35–90ºN as a reasonable domain for the reconstruction target. The final large-scale reconstruction was generated using a Gaussian process regression[52] and an autoregressive term[53] estimated from the observed temperature data (after they were transformed to logarithms). Error estimates (the 90% bounds) were produced by a Bayesian bootstrap algorithm[54], which generated 2000 emulations of the Gaussian process.

Group 15 reconstruction (R15). R15 applied no selection criteria on the nine raw TRW datasets, the correct cross-dating done by their providers was verified with some randomly chosen samples. Before detrending, TRW series of less than 50 rings were excluded from the dataset to avoid a potential

adverse influence on the calculation of the regional curve in the RCS process, since pith offset information was not available. R15 developed nine individual TRW chronologies for the different regional datasets. First, a data-adaptive power transformation was applied on the raw data to reduce the influence of outliers. To avoid potential bias in applying any kind of RC curve with negative slope, TRW data showing no significant slope or an even positive long-term trend (assuming that a positive slope cannot be related to an age-related signal) with increasing tree age, R15 separated all those individual series and detrended them by subtracting the mean of the original values in ARSTAN V44h3 software version[6]. If a positive juvenile growth trend was present only during the first 20 years of growth, R15 removed this juvenile phase from the data. In doing so, the developed RCs showed a monotonic decline, which helped to avoid possible artefacts by detrending with RCs with complex shapes. Subsequently, R15 applied a signal-free RCS on all datasets, residuals were calculated from the RC after power transformation, and the RC was smoothed with a length-adaptive -10y cubic spline using the software RCSigFree V45_v2b. In doing so, R15 did not differentiate between series from living and relict trees. In case that the RC showed a turning point with increasing trend in the younger section, R15 cut this increasing part of the RC off and used a straight by elongating the RC with the lowest value before the turning point. This turning point was reached at different tree ages in the different datasets (varying from 300–600 years), depending on the mean segment length of the included series. Before calculating the chronologies by calculating bi-weight robust means, R15 again merged the RCS-detrended and straight-line detrended series of each dataset in dplR package[55]. Since R15 had no local or regional climate data at hand, it correlated the nine regional chronologies against 0.5° × 0.5° CRU TS4.04 grid cells[51]. For each regional chronology, R15 computed regional means of climate data for the same spatial realm represented by the TRW data. This grid points included in these spatial windows varied from 1 (chronology SCO) to 1600 (chronology NYA). TRW data were correlated against monthly means of temperature and precipitation using a time window including all months of the growth year and the year prior to growth. Beside monthly data, also different combinations of seasonal averages were tested. To be able to calculate a north-hemispheric mean

temperature reconstruction, R15 selected a seasonal window as reconstruction target that showed high correlations between TRW data and temperature over all sites. Although highest mean correlations were found for the short summer season July–August ($r = 0.41$), R15 decided to reconstruct the June–October season, which showed only slightly lower mean correlations ($r = 0.38$), but represented a considerably longer period. R15 was well aware that in doing so, R15 lost some percent of explained variance in the regional climate reconstructions, since for some chronologies this was not the season showing the highest correlation with temperature data. After trying different time windows, R15 decided to compute proxy-target correlations over the period 1901 to the last year of the individual regional chronology. The chronology error was estimated as the bootstrapped standard error (± 2 SE) of the interannual TRI variation and the calibration error is calculated as the RMSE of the validation (± 2 RMSE). Afterwards, the uncertainty values of the chronology and calibration error were combined as the RMSE for the reconstruction[56]. Since the Siberian TRW chronology from Taimyr (TAI, *Larix gmelinii*)[26] showed a negative trend of TRW during the mid-20th century, which might be related to site-specific factors or divergence, it showed no significant correlation with growing season temperatures. The same was applied for the NYA chronology[25]. Since R15 did not have relevant metadata about the TRW material to be able to explain the weak temperature signal in the chronologies, R15 decided to exclude these two datasets from any further analytical steps. All of the remaining seven ensemble medians (GTB, SCO, QUL, NSC, ALP, YAM, ALT), however, reveal significant positive correlation coefficients ranging from $r = 0.23$ ($p < 0.05$) to 0.61 ($p < 0.01$) with current-year June to October temperatures over the 1901–2002 CE period of proxy-target overlap. The resulting seven regional temperature reconstructions were computed using linear regression between the z-scored chronologies and the target temperature data, the robustness of the obtained reconstructions was validated by calculating the K-folded cross validation using the caret-package for the statistical language R. Finally, the regional reconstructions were scaled as temperature deviations relative to the 1961–90 CE June–October temperature mean of the respective grid window. To generate a NH temperature reconstruction for the past 2000 years, the seven regional chronologies were divided into

four latitudinal NH sectors over the band 35–70°N, similar to previous studies that used eight sectors (also four longitudinal sectors, but split into two latitudinal bands together covering 40–75°N)[49]. The regional sectors corresponding to western north America, Europe, and Asia were represented by two regional temperature reconstructions (GTB+SCO, NSC+ALP, YAM+ALT), while the eastern north American sector was only represented by QUL, respectively. The NH mean for a belt stretching from 35–70°N was calculated by weighting the four regional sectors equally, meaning that all individual reconstructions had a weight of 0.125 to the final NH reconstruction, except QUL having a weight of 0.25, since it represented one of the four regional sectors alone. Since the last year of Southern Colorado Plateau chronology (SCO)[13] in the western United States ended in CE 2002, R15 computed the composite hemispheric reconstruction for the period CE 1–2002. The error band of the reconstruction was calculated by the merging the RMSEs of the individual reconstructions with the same weights as their contribution to the hemispheric mean.

Supplementary Table 1. Reconstruction characteristics. Key information about the 15 ensemble reconstructions, which used different selection criteria and detrending methods and programmes of the raw TRW measurement series, different choices of dendro sites and climatological target seasons and datasets, as well as different calibration and verification methods (see main text and Methods for details and abbreviations).

	Series Removal	Detrending	Site Selection	Season	Target	Calibration	Period
R1	none	Ensemble RCS	7: QUL, NSC, ALP, YAM, TAI, ALT, NYA	JJA	1950-2010, NH 30-90°, CRUTS4.04/Berkeley	scaling	1-2010
R2	none	SF RCS	9: GTB, SCO, QUL, NSC, ALP, YAM, TAI, ALT, NYA	JJA	1880-1980, NH 35-75°, CRUTS4.04	scaling	1-2015
R3	none	SF RCS	9: GTB, SCO, QUL, NSC, ALP, YAM, TAI, ALT, NYA	MJJAS	1880-2002, NH 30-75°, CRUTEM4.6	nested PCR	1-2016
R4	series removal (r <0.2)	SF RCS	7: GTB, SCO, QUL, NSC, ALP, YAM, ALT	JJA	1904-2014, reg mean , CRUTS4.04	regression	1-2016
R5	short series removal	SF RCS	9: GTB, SCO, QUL, NSC, ALP, YAM, TAI, ALT, NYA	JJ	1901-2000, NH 0-90°, CRUTEM4.6	PCR	1-2000
R6	none	SF RCS	7: QUL, NSC, ALP, YAM, TAI, ALT, NYA	JJA	1901-2010, reg mean, CRUTS4.04	regression	1-2010
R7	none	RCS	5: GTB, NSC, ALP, YAM, ALT	MJJAS	1900-2010, NH 30-90°, CRUTEM4.6.0	nested PCR	1-2010
R8	series removal (<100 yrs)	ARGC	9: GTB, SCO, QUL, NSC, ALP, YAM, TAI, ALT, NYA	JJA	1850-2020, NH 30-90°, CRUTEM4.6.0	AFR	1-2002
R9	none	Neg Expo	9: GTB, SCO, QUL, NSC, ALP, YAM, TAI, ALT, NYA	JJA	1801-2002, NH 40-90°, Berkeley	nested PCR	1-2016
R10	random to 200 series per site	2/3 spline	9: GTB, SCO, QUL, NSC, ALP, YAM, TAI, ALT, NYA	JJA	1750-1920, reg mean, Berkeley	scaling & splicing	1-2016
R11	duplicate removal	SF RCS	9: GTB, SCO, QUL, NSC, ALP, YAM, TAI, ALT, NYA	JJA	1850-2010, NH 30-70°, CRUTEM4.6	scaling	1-2010
R12	none	ABD	9: GTB, SCO, QUL, NSC, ALP, YAM, TAI, ALT, NYA	JJA	1901-2002, NH 15-65°, HadCRUT	regression	72-2002
R13	none	RCS	9: GTB, SCO, QUL, NSC, ALP, YAM, TAI, ALT, NYA	JJA	1850-2010, NH 40-75°, CRUTEM4.6	nested CPS	1-2009
R14	none	SF RCS	7: GTB, QUL, NSC, ALP, YAM, TAI, ALT	JJA	1901-2016, NH 35-90°, CRUTS4.1	GPR	1-2009
R15	series remoal (<50 yrs)	mean & SF RCS	7: GTB, SCO, QUL, NSC, ALP, YAM, ALT	JJASO	1901-2015, reg mean, CRUTS4.4	nested CPS	1-2002

Supplementary Table 2. Calibration/verification statistics. Full and split period, linear model calibration (Cal) and verification (Ver) statistics of the ensemble reconstruction mean as predictor of large-scale NH summer temperature variability. All statistics are performed on the un-differenced and first-differenced (1Diff) transform of both the proxy and target data. In each experiment verification is performed over the data not used in the calibration. Statistics present the degree to which the calibrated models (again on un-differenced and first differenced data) are robust in estimating temperature as expressed by the performance of their associated Reduction of Error (RE) and Coefficient of Efficiency (CE). Positive values of RE and CE suggest the models have predictive skill. Each column represents a different measure of interaction between the climate target and proxy variable along with, where appropriate, the probability (Pct) of obtaining that value by chance alone, the exceptions being RE, and CE. The four measures are, the Pearson, Robust Pearson, and Spearman correlations, and the statistical significance of the Cross Product (Xprod) between X and Y (Corr = correlation, Med = Median, tstat = *t*-statistic).

**Early Period (1794-1905)**	***Calibration Results (undifferenced data)***								
	Pearson	Robust	Spearman	RE	CE	Med RE	Med CE	t stat	P ct
	0.668	0.681	0.673	0.435	0.435	0.433	0.430	5.302	0.000
	Calibration Results (1st-differenced data)								
	Pearson	Robust	Spearman	RE	CE	Med RE	Med CE	t stat	P ct
	0.463	0.534	0.505	0.214	0.214	0.223	0.212	4.581	0.000
	Verification Results (undifferenced data)								
	Pearson	Robust	Spearman	RE	CE	Med RE	Med CE	t stat	P ct
	0.731	0.735	0.713	0.737	0.432	0.736	0.427	5.574	0.000
	Verification Results (1st-differenced data)								
	Pearson	Robust	Spearman	RE	CE	Med RE	Med CE	t stat	P ct
	0.539	0.575	0.555	0.256	0.254	0.260	0.253	4.106	0.000
**Late Period (1906-2015)**	***Calibration Results (undifferenced data)***								
	Pearson	Robust	Spearman	RE	CE	Med RE	Med CE	t stat	P ct
	0.752	0.738	0.715	0.447	0.447	0.447	0.444	5.004	0.000
	Calibration Results (1st-differenced data)								
	Pearson	Robust	Spearman	RE	CE	Med RE	Med CE	t stat	P ct
	0.539	0.577	0.554	0.290	0.290	0.287	0.278	4.120	0.000
	Verification Results (undifferenced data)								
	Pearson	Robust	Spearman	RE	CE	Med RE	Med CE	t stat	P ct
	0.676	0.681	0.681	0.608	0.232	0.608	0.229	4.009	0.000
	Verification Results (1st-differenced data)								
	Pearson	Robust	Spearman	RE	CE	Med RE	Med CE	t stat	P ct
	0.463	0.534	0.505	0.203	0.203	0.210	0.200	4.581	0.000

Supplementary Table 3. Summer temperature extremes. The five warmest and coldest reconstructed temperature anomalies in °C.

	R1	*R2*	*R3*	*R4*	*R5*	*R6*	*R7*	*R8*	*R9*	*R10*	*R11*	*R12*	*R13*	*R14*	*R15*	*Mean*	*Med*
**Five Warmest Summers**	990	246	2005	2012	406	2010	2010	11	2006	2012	1994	2001	1061	2005	613	2012	2012
	(1.22)	(0.44)	(0.65)	(0.75)	(0.62)	(0.56)	(0.79)	(1.21)	(1.14)	(1.67)	(0.57)	(0.39)	(1.16)	(0.84)	(0.59)	(0.84)	(0.75)
	287	1982	2006	2013	1160	1945	1998	2002	1998	2016	1982	1994	764	2006	679	2013	2013
	(0.98)	(0.40)	(0.64)	(0.70)	(0.47)	(0.49)	(0.68)	(1.19)	(1.10)	(1.60)	(0.57)	(0.39)	(0.93)	(0.83)	(0.55)	(0.67)	(0.67)
	282	242	2012	1998	405	2009	2003	9	2003	2003	1998	1998	986	2009	612	2003	2005
	(0.91)	(0.39)	(0.60)	(0.68)	(0.44)	(0.49)	(0.65)	(1.10)	(1.10)	(1.44)	(0.55)	(0.34)	(0.92)	(0.83)	(0.54)	(0.66)	(0.57)
	284	1955	2013	2003	89	1938	2006	10	242	2015	2005	1988	1161	2007	672	2006	2003
	(0.89)	(0.38)	(0.60)	(0.56)	(0.42)	(0.49)	(0.63)	(1.09)	(1.02)	(1.42)	(0.55)	(0.32)	(0.89)	(0.82)	(0.54)	(0.62)	(0.56)
	1942	1953	2010	2014	827	1942	2004	1736	674	2013	2006	1982	1160	2008	26	2005	2014
	(0.84)	(0.38)	(0.59)	(0.55)	(0.42)	(0.48)	(0.61)	(1.01)	(0.96)	(1.32)	(0.54)	(0.32)	(0.88)	(0.80)	(0.52)	(0.58)	(0.55)
**Five Coldest Summers**	545	544	1602	543	627	1602	544	1122	543	1642	544	545	543	1465	544	627	1601
	(-1.63)	(-1.34)	(-1.36)	(-2.90)	(-0.98)	(-0.79)	(-1.17)	(-0.82)	(-1.20)	(-1.80)	(-1.14)	(-0.89)	(-1.91)	(-1.49)	(-0.83)	(-1.28)	(-1.20)
	1699	627	1468	546	687	1601	543	1820	546	627	546	546	537	1606	1601	546	543
	(-1.73)	(-1.39)	(-1.36)	(-2.98)	(-1.04)	(-0.81)	(-1.21)	(-0.82)	(-1.21)	(-1.87)	(-1.19)	(-0.89)	(-1.93)	(-1.56)	(-0.86)	(-1.28)	(-1.21)
	543	543	1601	1681	1641	545	546	1615	1602	537	543	1642	546	1605	536	543	546
	(-1.77)	(-1.40)	(-1.42)	(-3.04)	(-1.07)	(-0.82)	(-1.25)	(-0.83)	(-1.22)	(-1.88)	(-1.20)	(-0.89)	(-2.02)	(-1.61)	(-0.86)	(-1.30)	(-1.21)
	1602	536	545	536	1642	1699	536	1335	1820	545	545	1601	545	1602	543	545	545
	(-1.81)	(-1.44)	(-1.49)	(-3.36)	(-1.12)	(-0.82)	(-1.26)	(-0.90)	(-1.22)	(-1.89)	(-1.25)	(-0.90)	(-2.12)	(-1.69)	(-0.87)	(-1.37)	(-1.27)
	536	545	536	627	536	536	545	1348	545	536	536	536	536	1601	1602	536	536
	(-2.02)	(-1.50)	(-1.71)	(-3.61)	(-1.24)	(-0.89)	(-1.27)	(-0.97)	(-1.37)	(-2.58)	(-1.36)	(-1.06)	(-2.47)	(-1.70)	(-0.98)	(-1.54)	(-1.34)

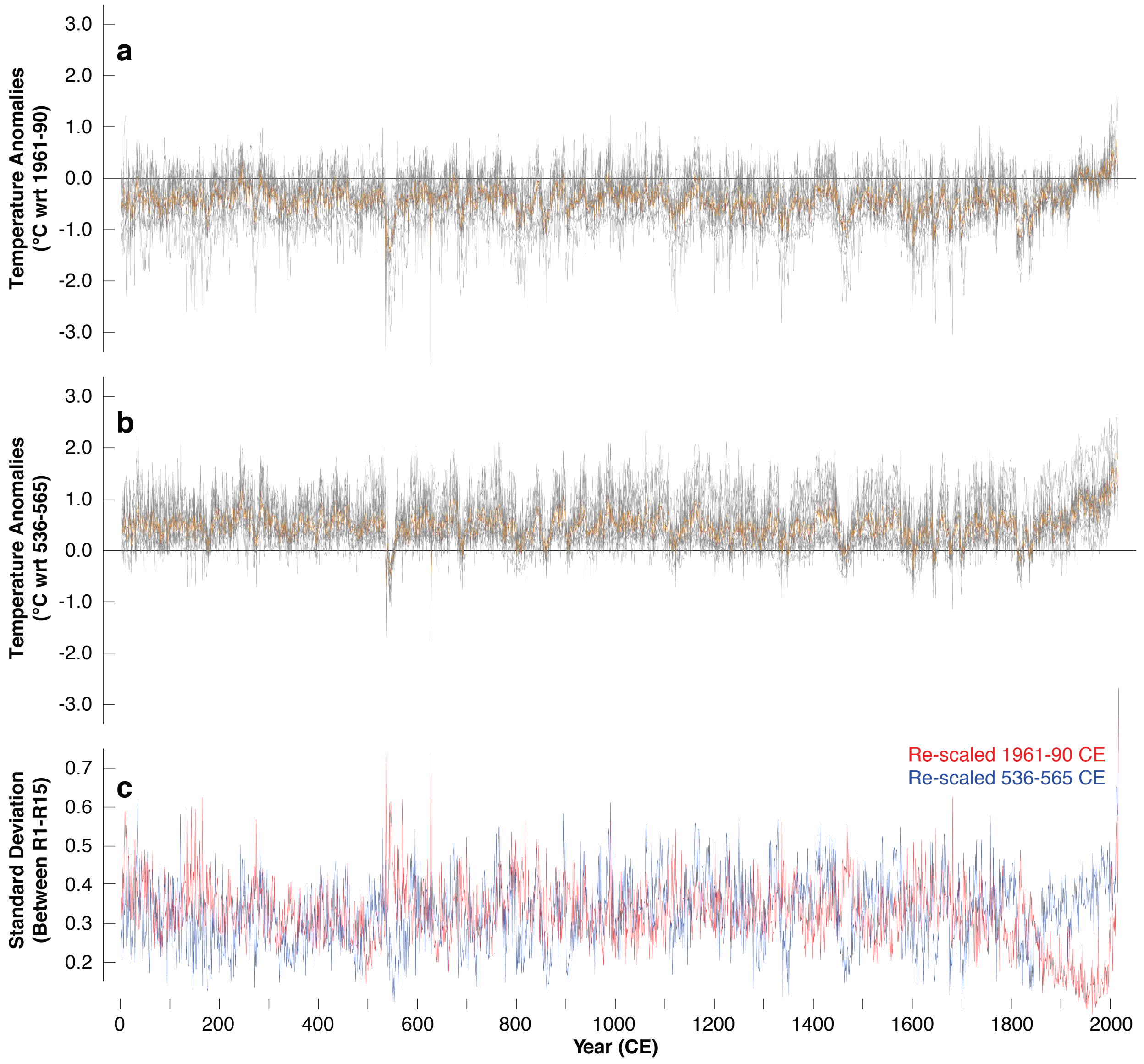

Supplementary Figure 1. Scaling bias. (**a**) The individual ensemble reconstructions (grey lines) and their mean and median (orange and red), scaled over 1961–1990 CE. (**b**) The individual ensemble reconstructions (grey lines) and their mean and median (orange and red), scaled over 536–565 CE. (**c**) The annual standard deviation values between all 15 ensemble reconstructions.

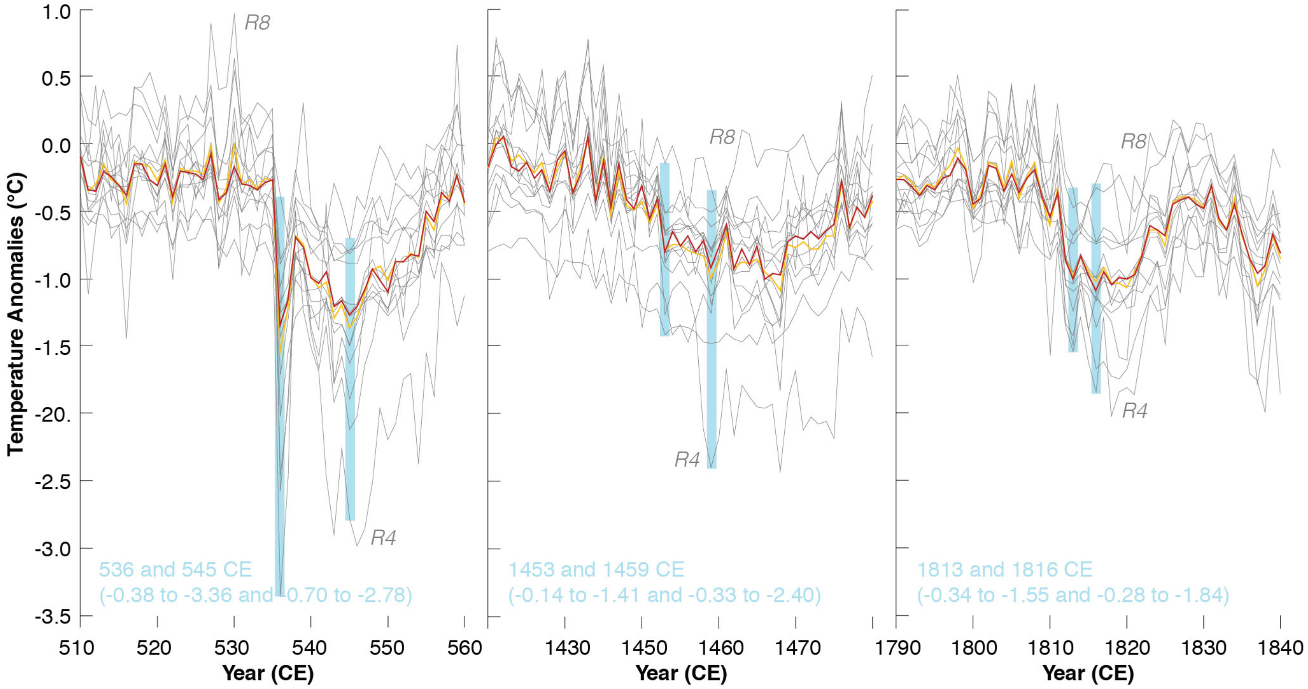

Supplementary Figure 2. Post-volcanic cooling. Behaviour of the 15 ensemble reconstructions (grey lines), together with their mean and median (orange and red) during the three most pronounced cold spells following volcanic eruptions.

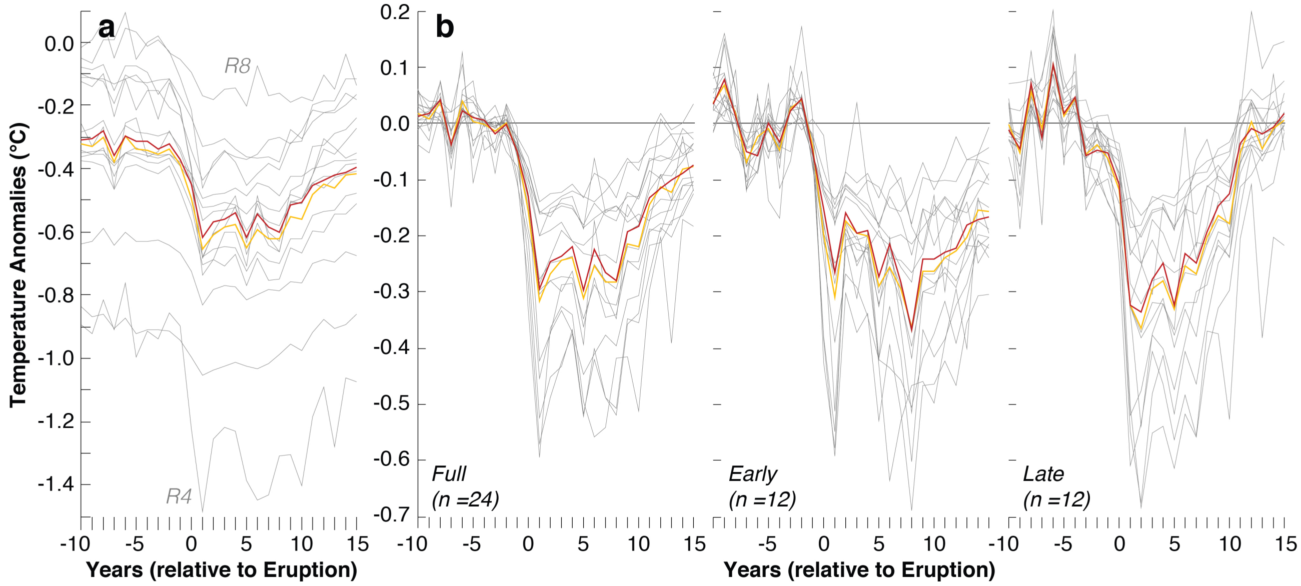

Supplementary Figure 3. Superposed Epoch Analysis. (**a**) Average response of the 15 ensemble reconstructions (grey), as well as their mean and median (orange and red) to the 24 strongest volcanic eruptions of the Common Era. (**b**) Post-volcanic cooling relative to 10-year periods before all 24 eruptions (full), as well as after splitting into two early/late sub-periods of 12 eruptions each (</> 1170 CE). All eruptions exceed the Stratospheric Sulfur Injection (SSI) of 1991 Pinatubo event and occurred in 169, 266, 304, 433, 536, 574, 626, 682, 817, 939, 1108, 1171, 1191, 1230, 1257, 1286, 1345, 1458, 1600, 1640, 1695, 1815, 1835, and 1883 CE.

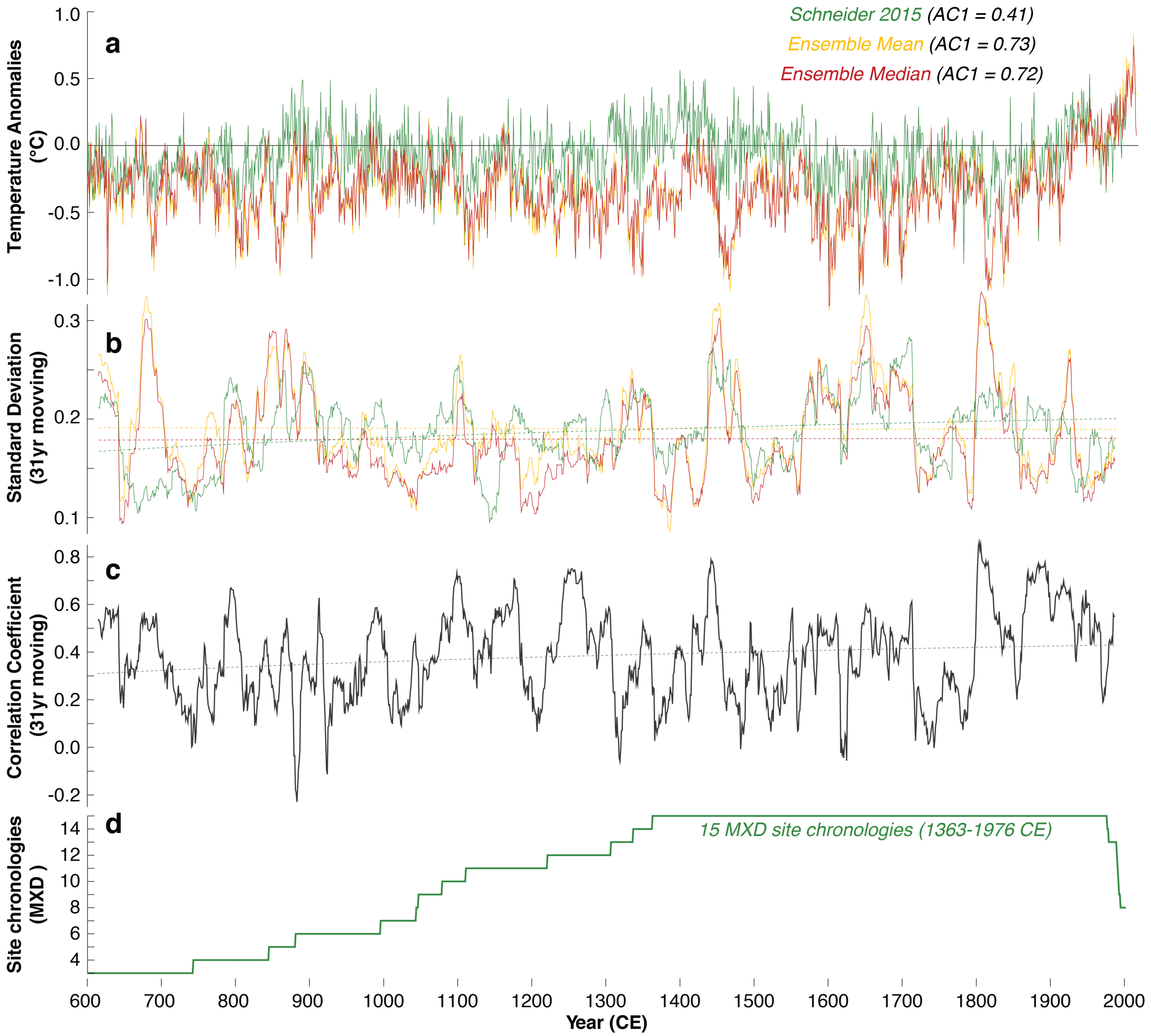

Supplementary Figure 4. Parameter comparison. (**a**) The mean and median ensemble reconstructions (orange and red), together with the MXD-based JJA large-scale NH reconstruction[57], which contains a substantially lower first-order autocorrelation coefficient (600–2002 CE). (**b**) Moving 31-year standard deviations of the mean and median ensemble reconstructions (orange and red), as well as the MXD-based reconstruction (green), with the dashed lines showing a long-term logarithmic variance decline in the MXD back in time. (**c**) Moving 31-year correlation coefficients between the ensemble mean and the MXD-based reconstruction, with the dashed line showing a long-term logarithmic coherency decline back in time. (**d**) Number of MXD site chronologies that are declining from 15 to three back in time.

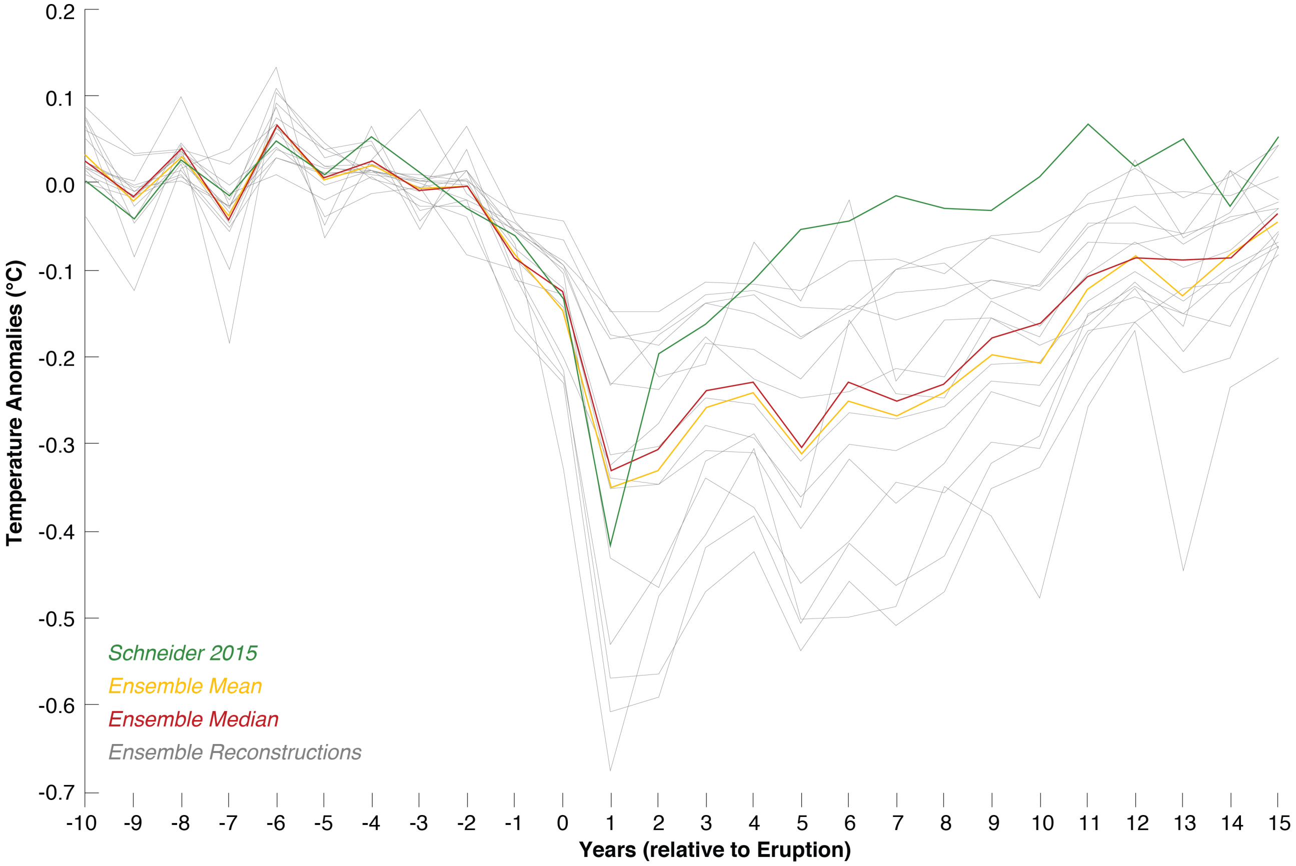

Supplementary Figure 5. Parameter response. Average response of the MXD-based JJA large-scale NH reconstruction (green)[57], as well as the 15 ensemble reconstructions (grey), and their mean and median (orange and red) to the 18 strongest volcanic eruptions back to 600 CE (expressed as anomalies with respect to the 10-year periods before the eruptions). All eruptions exceed the Stratospheric Sulfur Injection (SSI) of 1991 Pinatubo event and occurred in 626, 682, 817, 939, 1108, 1171, 1191, 1230, 1257, 1286, 1345, 1458, 1600, 1640, 1695, 1815, 1835, and 1883 CE.

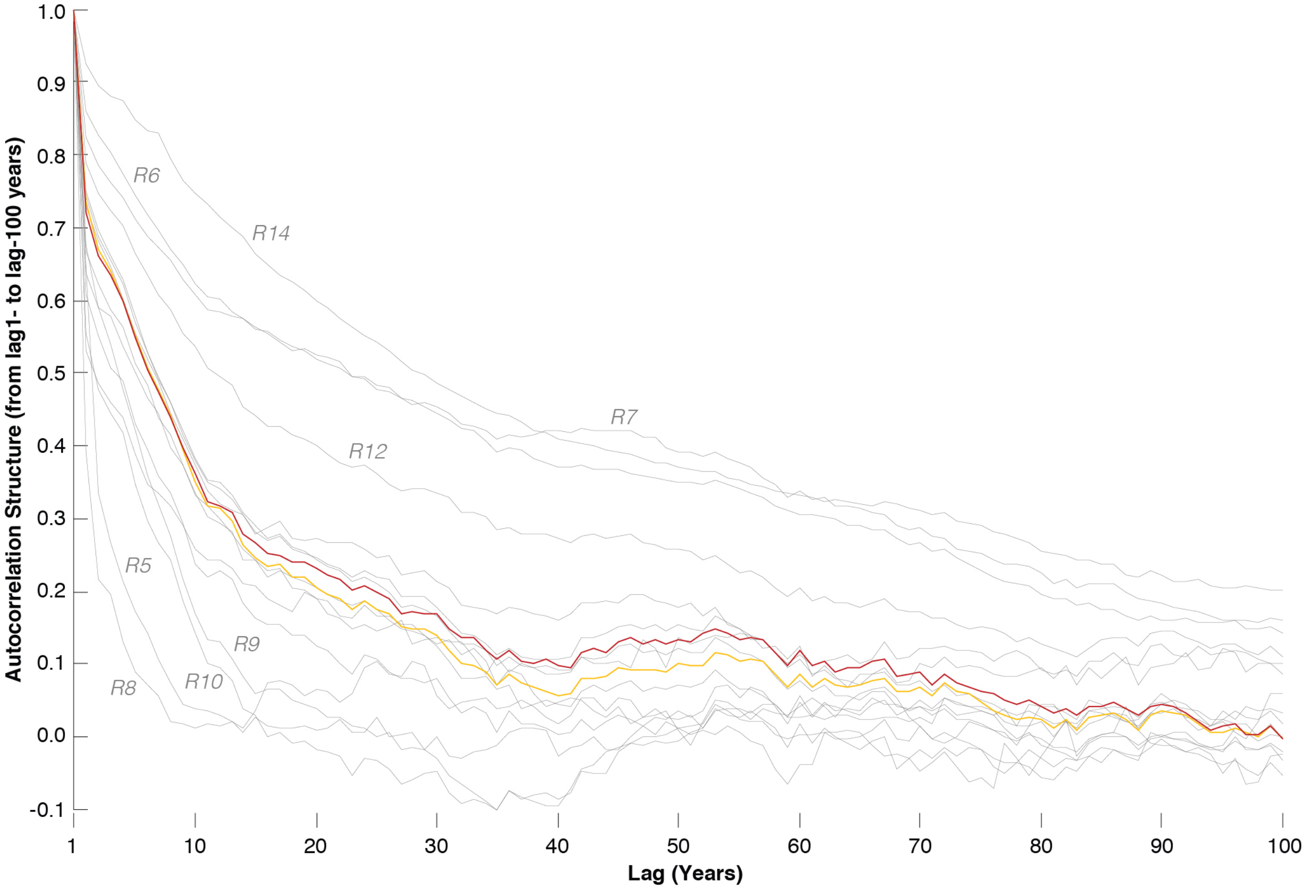

Supplementary Figure 6. Autocorrelation function. Correlation coefficients of the ensemble reconstructions (grey), as well as their mean and median (orange and red) against the same time-series after lagging by 1–100 years.

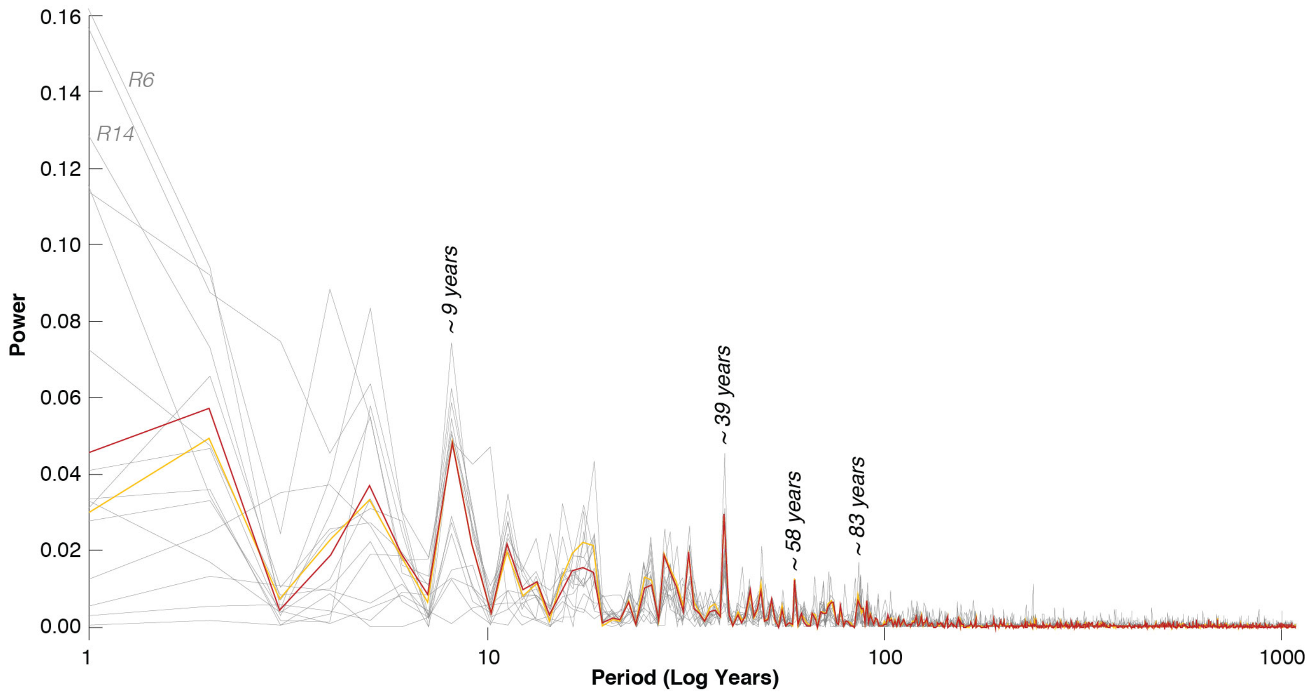

Supplementary Figure 7. Power spectra. Periodogram of the ensemble reconstructions (grey), as well as their mean and median (orange and red) computed over the individual time-series length between 1 and 2016 CE.

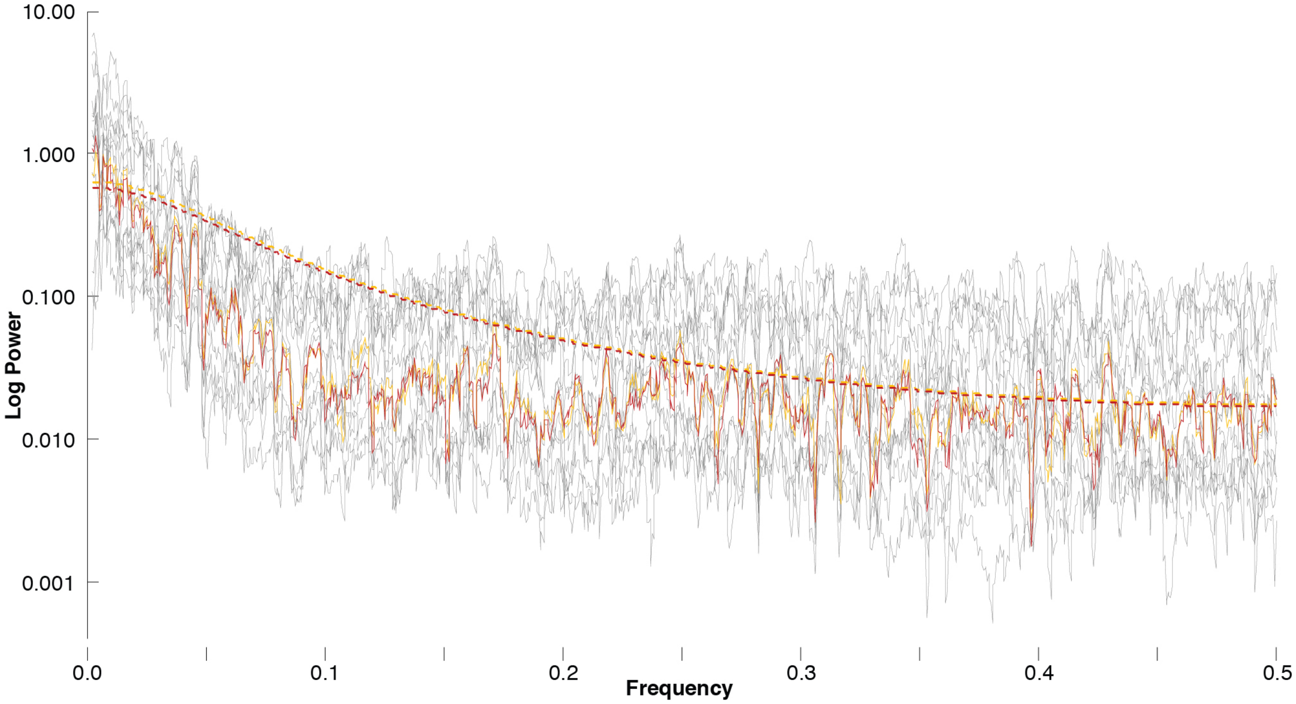

Supplementary Figure 8. Power spectra. Multi-Taper Method[58] of the ensemble reconstructions (grey), as well as their mean and median (orange and red) computed over the individual time-series length between 1 and 2016 CE (using 4-year resolution and seven tapers). Dashed lines are the 95% significance levels relative to estimated (AR1) background noise.

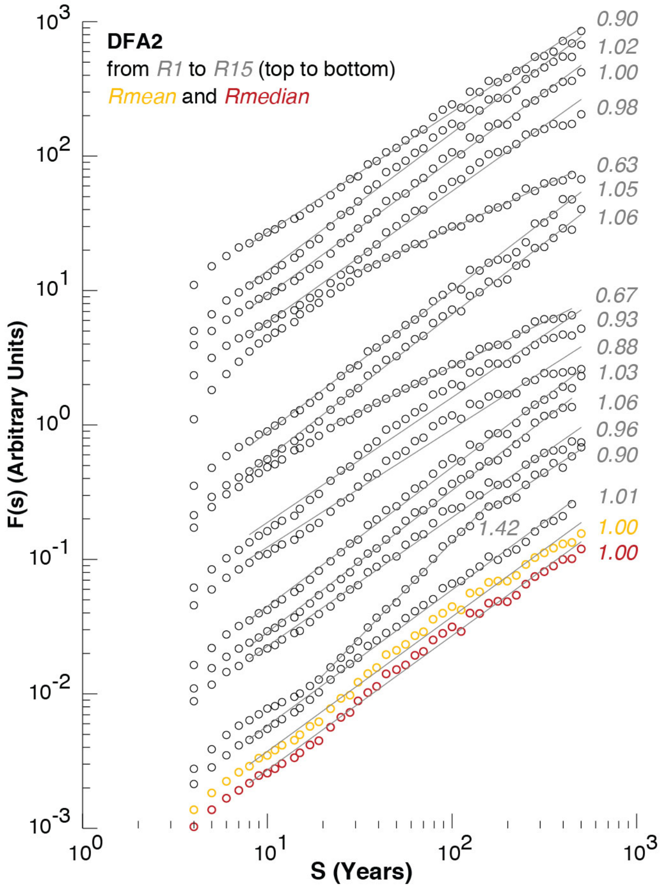

Supplementary Figure 9. Long-term persistence. DFA2 and WT2 fluctuation functions of the 15 ensemble reconstructions, their mean and median, and the most appropriate power-law (lines in the double logarithmic representation) fits to the data. The fluctuation functions of R1, R2, R3, R6, R7, R11, R12, R13, R15, Rmean and Rmedian can be well described by power-laws with Hurst exponents (H) between 0.90 and 1.06. These reconstructions posse a higher long-term persistence than one would expect based on observational records. The reconstruction R4, R5, R8, R9, R10 and R14 deviate from a single power-law behaviour. R5 shows a clear long-term persistent behaviour for the DFA2 and WT2 fluctuation functions with H = 0.63 on time scales > 8 years, though behaves different on shorter time scales. The long-term persistence of this reconstruction is lower than expected from the observational data. The WT2 fluctuation function of R8 shows a power-law behaviour with H = 0.73 over the full range, i.e., from 1–100 years. This Hurst exponent (H) is close to the observationally expected value. The DFA2 analysis of R8 shows H = 0.67 on time scales greater than 20 years, indicating a little less long-term persistence on longer time scales than the WT2 analysis suggests. R14 shows a long-term persistent behaviour with H = 0.90 (DFA2) and H = 0.83 (WT2) on time scales greater than about 40

years. On medium time scales between six and about 40 years the Hurst exponents are 1.42 (DFA2) and 1.44 (WT2). This is interesting because Hurst exponents as high as 1.42 or 1.44 are indicative of very strong persistence, and do not occur in any observational climate data, be it air or sea surface temperatures, precipitation totals, river run-off rates or sea ice extent. A Hurst exponent of 1.5 can be obtained if white noise data (H = 0.5) are summed up.

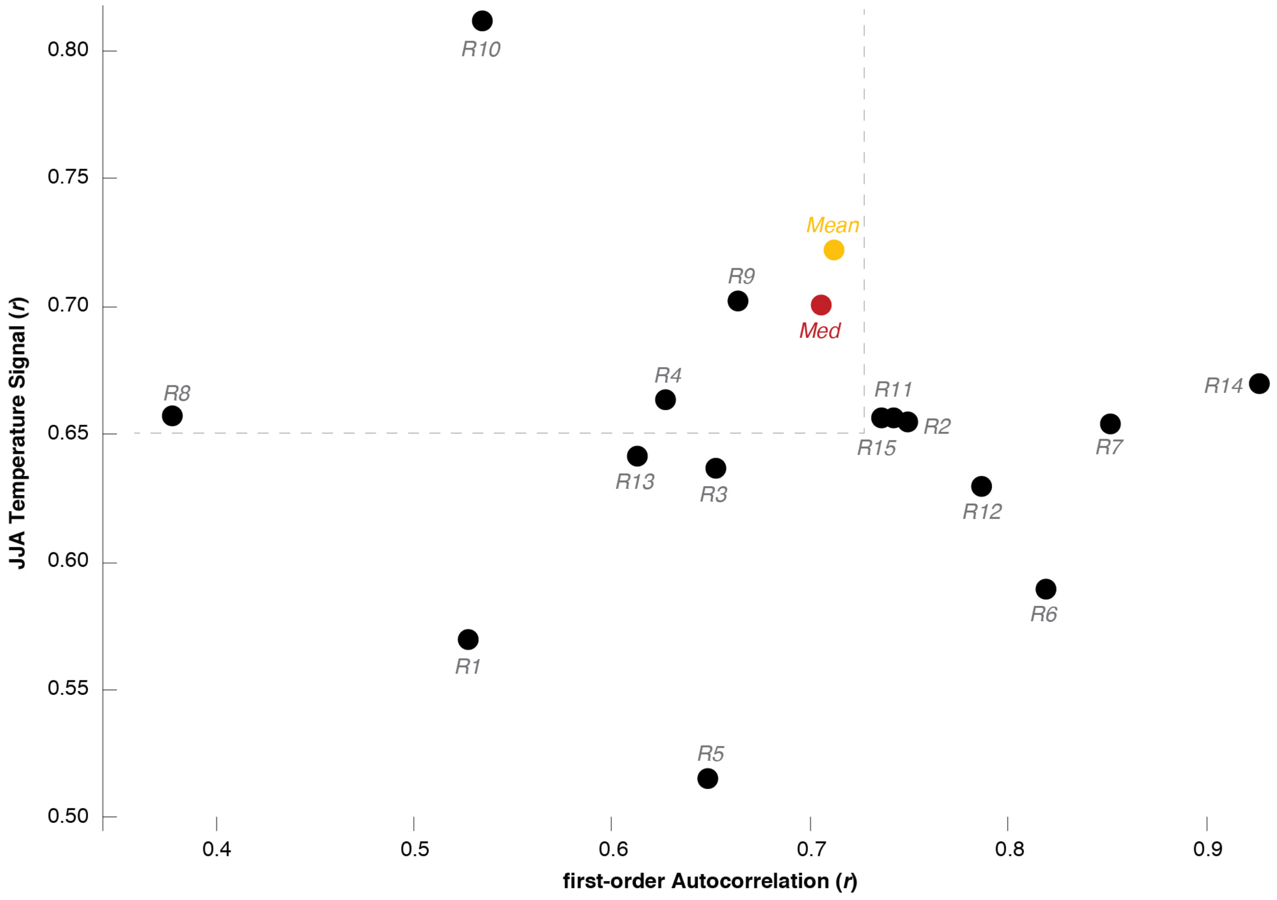

Supplementary Figure 10. Reconstruction characteristics. Visual comparison between the JJA summer temperature signal (r) and the first-order autocorrelation structure AC1 (r) of the 15 reconstructions and their mean and median. Reconstructions with a strong temperature signal and a low AC1 are considered more suitable (upper left), compared to those with low temperature sensitivity and high autocorrelation (bottom right). It should be noted that the exceptionally high temperature signal in R10 results from the splicing of instrumental data, i.e. the proxy is not independent from its target.

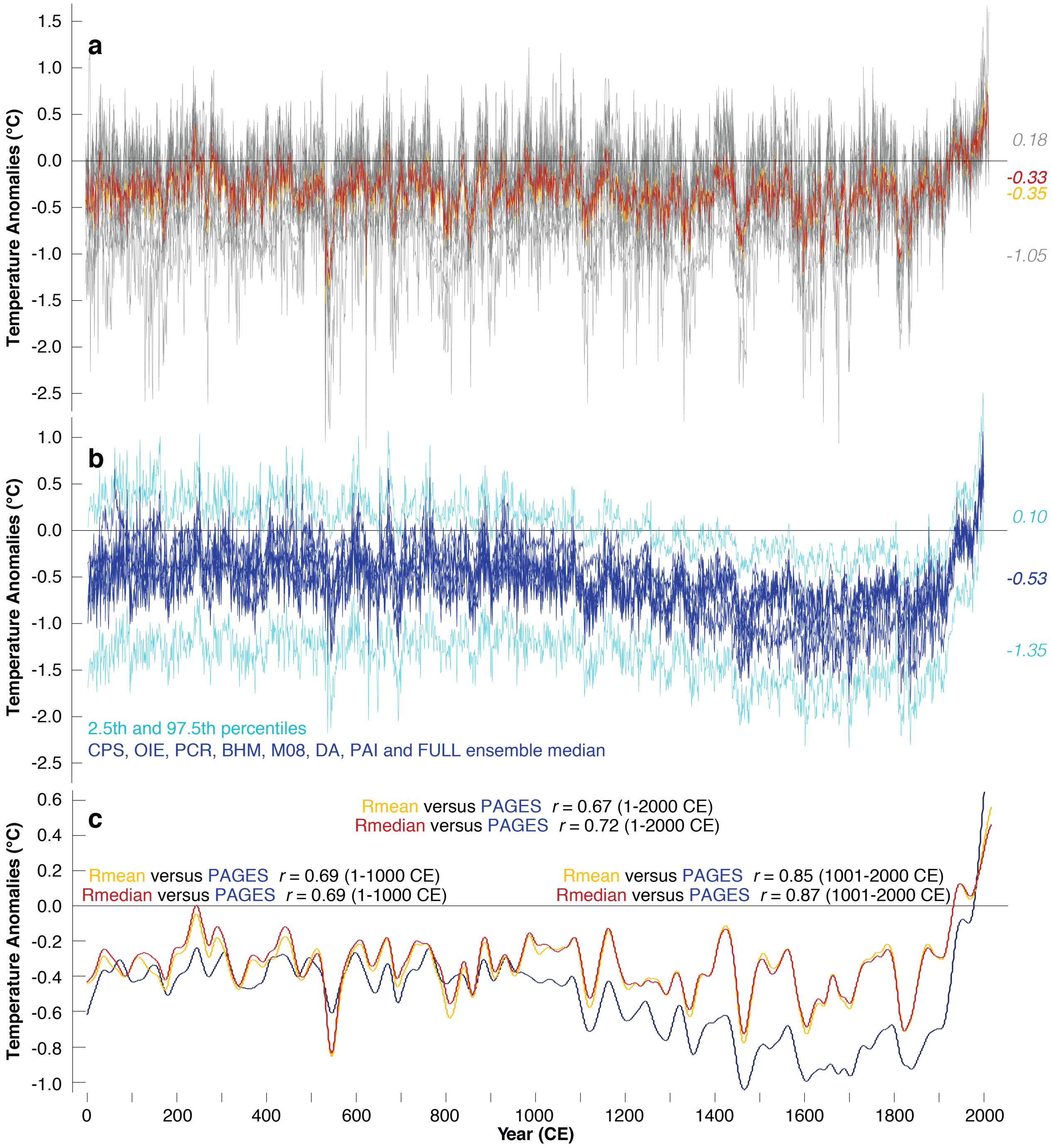

Supplementary Figure 11. Multi-proxy comparison. (**a**) The 15 ensemble reconstructions (grey lines), together with their mean and median (orange and red). (**b**) The eight PAGES19 products (following different reconstruction methods)[59], together with the 2.5th and 97.5th percentile confidence intervals from all ensemble reconstruction members between 1 and 2000 CE. (**c**) The mean and median (orange and red) of our 15 reconstructions, as well as the PAGES19 full ensemble median after 50-year low-pass filtering. All PAGES19 data[59] were rescaled against mean 30–70°N extra-tropical landmass JJA temperature anomalies relative to the 1961–90 CE.

Supplementary References

1. Esper, J., Cook, E.R., Krusic, P.J., Peters, K., Schweingruber, F.H., 2003. Tests of the RCS method for preserving low-frequency variability in long tree-ring chronologies. *Tree-Ring Res.* **59**, 81–98.
2. Melvin, T.M., Briffa, K.R., 2008. A "signal-free" approach to dendroclimatic standardisation. *Dendrochronologia* **26**, 71–86.
3. Melvin, T.M., Briffa, K.R., 2014. CRUST: software for the implementation of regional chronology standardisation: part 1. Signal-free RCS. *Dendrochronologia* **32**, 7–20.
4. Osborn, T.J., Briffa, K.B., Jones, P.D., 1997. Adjusting variance for sample size in tree-ring chronologies and other regional mean timeseries. *Dendrochronologia* **15**, 89–99.
5. Cook, E.R., Peters, K., 1997. Calculating unbiased tree-ring indices for the study of climatic and environmental change. *Holocene* **7**, 361–370.
6. Cook, E.R., Krusic, P.J., Peters, K., Holmes, R.L., 2017. *Program ARSTAN version48d2: Autoregressive tree-ring standardization program*. Tree-Ring Laboratory of LDEO.
7. Cook, E.R., 1985. *A time series analysis approach to tree-ring standardization*. PhD dissertation, University of Arizona, Tucson.
8. Büntgen, U., et al., 2020. Prominent role of volcanism in Common Era climate variability and human history. *Dendrochronologia* **64**, 125757.
9. Büntgen, U. Kaczka, R.J., Trnka, M., Rigling, A., 2012. Ensemble estimates reveal a complex hydroclimatic sensitivity of pine growth at Carpathian cliff sites. *Agricult. Forest Meteorol.* **160**, 100–109.
10. Jones, P.D., Lister, D.H., Osborn, T.J., Harpham, C., Salmon, M., Morice, C.P., 2012. Hemispheric and large-scale land-surface air temperature variations: an extensive revision and update to 2010. *J. Geophys. Res.* **117**, D05127.

11. Rhode, R., Muller, R., Jacobsen, R., Perlmutter, S., Rosenfeld, A., Wurtele, J., Curry, J., Wickham, C., Mosher, S., 2013. Berkeley Earth temperature averaging process. *Geoinformat. Geostat.* Overview **1**, 1.

12. Frank, D.C., Büntgen, U. Böhm, R., Maugeri, M., Esper, J., 2007. Warmer early instrumental measurements versus colder reconstructed temperatures: shooting at a moving target. *Quat. Sci. Rev.* **26**, 3298–3310.

13. Salzer, M.W., Bunn, A.G., Graham, N.E., Hughes, M.K., 2014. Five millennia of paleotemperature from tree-rings in the Great Basin, USA. *Clim. Dyn.* **42**, 1517–1526.

14. Salzer, M.W., Kipfmueller, K.F., 2005. Reconstructed temperature and precipitation on a millennial timescale from tree-rings in the Southern Colorado Plateau, U.S.A. *Clim. Change* **70**, 465–487.

15. Williams, A.P., Cook, E.R., Smerdon, J.E., Cook, B.I., Abatzoglou, J.T., Bolles, K., Baek, S.H., Badger, A.M., Livneh, B., 2020. Large contribution from anthropogenic warming to an emerging North American megadrought. *Science* **368**, 314–318.

16. Esper, J., Frank, D.C., Wilson, R.J.S., Briffa, K.R., 2005. Effect of scaling and regression on reconstructed temperature amplitude for the past millennium. *Geophys. Res. Lett.* **32**, L07711.

17. Wigley, T.M.L., Briffa, K.R., Jones, P.D., 1984. On the average value of correlated time series, with applications in dendroclimatology and hydrometeorology. *J. Clim. Appl. Meteorol.* **23**, 201–213.

18. Briffa, K.R., Jones, P.D., Bartholin, T.S., Eckstein, D., Schweingruber, F.H., Karlén, W., Zetterberg, P., Eronen, M., 1992. Fennoscandian summers from ad 500: temperature changes on short and long timescales. *Clim. Dyn.* **7**, 111–119.

19. Briffa, K.R., Melvin, T.M., 2011. *A closer look at Regional Curve Standardization of tree-ring records: justification of the need, a warning of some pitfalls, and suggested improvements in its application.* In: M.K. Hughes, T.W. Swetnam and H.F. Diaz (Editors), Dendroclimatology: Progress and Prospects. Springer Netherlands.

20. Yang, B., Qin, C., Wang, J., He, M., Melvin, T.M., Osborn, T.J., Briffa, K.R., 2014. A 3,500-year tree-ring record of annual precipitation on the northeastern Tibetan Plateau. *Proc. Natl. Acad. Sci. U.S.A.* **111**, 2903–2908.

21. Cook, E.R., D'Arrigo, R.D., Mann, M.E., 2002. A well-verified, multiproxy reconstruction of the winter North Atlantic Oscillation index since A.D. 1400. *J. Clim.* **15**, 1754–1764.

22. Luterbacher, J., et al., 2016. European summer temperatures since Roman times. *Environ. Res. Lett.* **11**, 024001.

23. Wang, J., Yang, B., Ljungqvist, F.C., 2020. Moisture and temperature covariability over the Southeastern Tibetan Plateau during the past nine centuries. *J. Clim.* **33**, 6583–6598.

24. Cook, E.R., Meko, D.M., Stahle, D.W., Cleaveland, M.K., 1999. Drought reconstructions for the continental United States. *J. Clim.* **12**, 1145–1162.

25. Churakova (Sidorova), O.V., et al., 2019. Siberian tree-ring and stable isotope proxies as indicators of temperature and moisture changes after major stratospheric volcanic eruptions *Clim. Past* **15**, 685–700.

26. Naurzbaev, M.M., Vaganov, E.A., Sidorova, O.V., Schweingruber, F.H., 2002. Summer temperatures in eastern Taimyr inferred from a 2427-year late-Holocene tree-ring chronology and earlier floating series. *Holocene* **12**, 727–736.

27. Büntgen, U., Tegel, W., Nicolussi, K., McCormick, M., Frank, D., Trouet, V., Kaplan, J., Herzig, F., Heussner, U., Wanner, H., Luterbacher, J., Esper, J., 2011. 2500 years of European climate variability and human susceptibility. *Science* **331**, 578–582.

28. Büntgen, U., Myglan, V.S., Ljungqvist, F.C., McCormick, M., Di Cosmo, N., Sigl, M., Jungclaus, J., Wagner, S., Krusic, P.J., Esper, J., Kaplan, J.O., de Vaan M.A.C., Luterbacher, J., Wacker, L., Tegel, W., Kirdyanov, A.V., 2016. Cooling and societal change during the Late Antique Little Ice Age from 536 to around 660 AD. *Nat. Geosci.* **9**, 231–236.

29. Gennaretti, F., Arseneault, D., Nicault, A., Perreault, L., Bégin, Y., 2014. Volcano-induced regime shifts in millennial tree-ring chronologies from northeastern North America. *Proc. Natl. Acad. Sci. U.S.A.* **111**, 10077–10082.

30. Melvin, T.M., Grudd, H., Briffa, K.R., 2012. Potential bias in 'updating' tree-ring chronologies using regional curve standardisation: Re-processing 1500 years of Torneträsk density and ring-width data. *Holocene* **23**, 364–373.

31. Bunn, A.G., 2010. Statistical and visual crossdating in R using the dplR library. *Dendrochronologia* **28**, 251–258.

32. Buras, A., 2017. A comment on the expressed population signal. *Dendrochronologia* **44**, 130–132.

33. Wilson, R., Cook, E., D'Arrigo, R., Riedwyl, N., Evans, M.N., Tudhope, A., Allan, R., 2010. Reconstructing ENSO: the influence of method, proxy data, climate forcing and teleconnections. *J. Quat. Sci.* **25**, 62–78.

34. Cook, E.R., Briffa, K.R., Jones, P.D., 1994. Spatial regression methods in dendroclimatology: A review and comparison of two techniques. *Int. J. Climatol.* **14**, 379–402.

35. Revelle, W., 2017. *psych: Procedures for Personality and Psychological Research, Version = 1.7.8* edn., Northwestern University, Evanston, Illinois, USA.

36. Cook, E.R., Kairiukstis, L.A., 1990. *Methods of dendrochronology: applications in the environmental sciences.* Kluwer Academic Publishers, Boston.

37. Esper, J., Frank, D., Büntgen, U., Verstege, A., Luterbacher, J., Xoplaki, E., 2007. Long-term drought severity variations in Morocco. *Geophys. Res. Lett.* **34**, L17702.

38. Nicault, A., Guiot, J., Edouard, J.-L., Brewer, S., 2010. Preserving long-term fluctuations in standardisation of tree-ring series by the adaptive regional growth curve (ARGC) and its validation with southern alps pdsi reconstruction. *Dendrochronologia* **28**, 1–12.

39. Guiot, J., Corona, C., ESCARSEL members, 2010. Growing season temperatures in Europe and climate forcings over the past 1400 years. *PLoS One* **5**, e9972.

40. Morice, C.P., Kennedy, J.J., Rayner, N.A., Jones, P.D., 2012. Quantifying uncertainties in global and regional temperature change using an ensemble of observational estimates: The HadCRUT4 data set. *J. Geophys. Res.* **117**, D08101.

41. Briffa, K.R., Osborn, T.J., Schweingruber, F.H., Harris, I.C., Jones, P.D., Shiyatov, S.G., Vaganov, E., 2001. Low-frequency temperature variations from a northern tree ring density network. *J. Geophys. Res.* **106**, 2929–2941.

42. Brohan, P., Kennedy, J.J., Harris, I., Tett, S.F.B., Jones, P.D., 2006. Uncertainty estimates in regional and global observed temperature changes: a new dataset from 1850. *J. Geophys. Res.* **111**, D12106.

43. Jones, P.D., New, M., Parker, D.E., Martin, S., Rigor, I.G., 1999. Surface air temperature and its variations over the last 150 years. *Rev. Geophys.* **37**, 173–199.

44. Hantemirov, R.M., Shiyatov, S.G., 2002. A continuous multimillennial ring-width chronology in Yamal, northwestern Siberia. *Holocene* **12**, 717–726.

45. Osborn, T.J., Jones, P.D. 2014. The CRUTEM4 land-surface air temperature data set: construction, previous versions and dissemination via Google Earth. *Earth Syst. Sci. Data* **6**, 61–68.

46. Cowtan, K., Way, R.G., 2014. Coverage bias in the HadCRUT4 temperature series and its impact on recent temperature trends. *Q. J. R. Meteorol. Soc.* **140**, 1935e1944.

47. LaMarche, V.C. Jr., Stockton, C.W. 1974. Chronologies from temperature-sensitive bristlecone pines at upper treeline in western United States. *Tree-Ring Bull.* **34**, 21–45.

48. Hughes, M.K., Funkhouser, G., 2003. Frequency-Dependent Climate Signal in Upper and Lower Forest Border Tree Rings in the Mountains of the Great Basin. *Clim. Change* **56**, 233–244.

49. Wilson, R.J.S., et al., 2016. Last millennium northern hemisphere summer temperatures from tree rings: Part I: The long-term context. *Quat. Sci. Rev.* **134**, 1–18.

50. Tierney, J.E., Abram, N.J., Anchukaitis, K.J., Evans, M.N., Giry, C., Kilbourne, K.H., Saenger, C.P., Wu, H.C., Zinke, J., 2015. Tropical sea surface temperatures for the past four centuries reconstructed from coral archives. *Paleoceanography* **30**, 226e252.

51. Harris, I., Osborn, T.J., Jones, P., Lister, D., 2020. Version 4 of the CRU TS monthly high-resolution gridded multivariate climate dataset. *Nat. Sci. Data* **7**, 109.

52. Barber, D., 2012. *Bayesian Reasoning and Machine Learning*. Cambridge University Press.

53. Shumway, R.H., Stoffer, D.S., 2017. *Time Series Analysis and Its Applications: With R examples*. Springer. Vancouver.

54. Shao, J., Tu, D., 2012. *The Jackknife and Bootstrap*. Springer Science & Business Media.

55. Bunn, A.G., 2008. A dendrochronology program library in R (dplR). *Dendrochronologia* **26**, 115–124.

56. Meier, W.J.-H., Aravena, J.-C., Jana, R., Braun, M.H., Hochreither, P., Soto-Rogel, P., Griessinger, J., 2020. A tree-ring $\delta^{18}O$ series from southernmost Fuego-Patagonia is recording flavours of the Antarctic Oscillation. *Glob. Plan. Change* **195**, 103302.

57. Schneider, L., Smerdon, J.E., Büntgen, U., Wilson, R.J.S., Myglan, V.S., Kirdyanov, A.V., Esper, J., 2015. Revising midlatitude summer temperatures back to A.D. 600 based on a wood density network. *Geophys. Res. Lett.* **42**, 4556–4562.

58. Thomson, D.J., 1982. Spectrum **estimation** and harmonic analysis. *Proc. IEEE* **70**, 1055–1096.

59. PAGES2k Consortium, 2019. Consistent multidecadal variability in global temperature reconstructions and simulations over the Common Era. *Nat. Geosci.* **12**, 643–649.
